# Origin of a self‐compatibility associated MITE in *Petota* and its application in hybrid potato breeding

**DOI:** 10.1111/nph.70093

**Published:** 2025-03-31

**Authors:** Saihang Zhang, Qinggang Liao, Zhan Zhang, Xu Zhu, Yuxin Jia, Yi Shang, Ling Ma

**Affiliations:** ^1^ Key Laboratory for Potato Biology of Yunnan Province, The CAAS‐YNNU‐YINMORE Joint Academy of Potato Science Yunnan Normal University Kunming 650500 China; ^2^ College of Horticulture Nanjing Agricultural University Nanjing 210095 China; ^3^ Yunnan International Joint R&D Center for Sustainable Development and Utilization of Biological Resources Kunming 650500 China

**Keywords:** diploid hybrid breeding, miniature inverted‐repeat transposable element, self‐incompatibility, *S‐locus inhibitor (Sli)*, true potato seeds

## Abstract

- Hybrid potato breeding offers a promising solution to tackle the challenges in potato breeding. However, most diploids are self‐incompatible (SI), which hinders the development of inbred lines. *S‐locus inhibitor* (*Sli*) is a ‘master key’ gene capable of conferring self‐compatibility (SC) to most of the SI diploids, yet the regulatory mechanism underlying its male gamete‐specific expression remains unclear, limiting its breeding potential.
- This study has uncovered that a miniature inverted‐repeat transposable element (Mi‐549) within the *Sli* promoter can affect the methylation pattern of the promoter, thereby regulating the pollen‐specific expression of *Sli* as well as the SC phenotype in diploids.
- We delved into the origin of Mi‐549 within *Petota* and found that Mi‐549 was likely acquired fortuitously in wild *Solanum lesteri* but was not favored during domestication, probably due to the asexual propagation nature of potato.
- Although Mi‐549 and its impacts on *Sli* as well as SC are not selected, screening of Mi‐549 identified three novel SC accessions that belong to *S. lesteri*, *Solanum neocardenasii* and *Solanum stenotomum*, which enrich the germplasm pool associated with stress and pest resistance and hold significant value for breeding applications.

## Introduction

Potato (*Solanum tuberosum* L.), a staple crop consumed by over 1.3 billion people spanning > 150 countries world‐wide, holds paramount significance for global food security (Stokstad, 2019). Currently, commercial potato cultivars are mainly derived from a narrow tetraploid subgroup, *S*. *tuberosum* Group *stenotomum*, which offers advantages such as large tubers and high yield (Spooner *et al*., 2014; Hardigan *et al*., 2017). However, there are challenges for traditional tetraploid potato breeding, including genetic variation loss because of the bottleneck effect and prolonged breeding cycles due to complex tetraploid genetics (Zhang *et al*., 2021; Kardile *et al*., 2022). Diploid hybrid breeding, using true seeds instead of tubers for propagation, exhibits a much higher reproductive and breeding efficiency than tetraploid breeding. Additionally, *c*. 70% of the naturally tuber‐bearing *Solanum* section *Petota*, comprising over 100 species, are diploid, offering a rich genetic resource for breeding (Jansky *et al*., 2016; Wu *et al*., 2023). However, self‐incompatibility (SI) is observed in most diploids, which hinders the creation of inbred lines essential for hybrid breeding (Ma *et al*., 2021; Zhang *et al*., 2021).

Potato exhibits gametophytic SI controlled by the *S*‐locus consisting of pistil‐specific *S‐RNase* and a group of pollen‐specific *S‐locus F‐box* (*SLF*) genes (Zhang *et al*., 2009). Generally, SLF interacts weakly with self*S*‐RNase but strongly with nonself* S*‐RNase, thereby preventing self‐fertilization and ensuring SI (Qiao *et al*., 2004; Hua & Kao, 2006; Kubo *et al*., 2010; Zhao *et al*., 2022). Therefore, self‐compatibility (SC) diploid potatoes can be achieved by knocking out self *S‐RNase*, selecting naturally occurring low‐expressed *S‐RNase* alleles, or overexpressing nonselffunctional *SLFs* (Ye *et al*., 2018; Zhang *et al*., 2021; Zhao *et al*., 2022). Notably, a *nonS‐locus* F‐box gene *S*‐locus inhibitor (*Sli*) was identified from naturally SC genotypes such as chc525‐3 and RH89‐039‐16 (RH). Sli can interact with multiple self and nonself*S*‐RNases to overcome SI across different genotypes and thus plays a key role in diploid potato breeding (Hosaka & Hanneman, 1998; Peterson *et al*., 2016; Clot *et al*., 2020; Eggers *et al*., 2021; Ma *et al*., 2021). Interestingly, the *Sli* promoter contains a 549 bp miniature inverted transposon element (MITE) insertion (named as Mi‐549 hereafter) (Chen *et al*., 2014). Miniature inverted transposon elements are known to suppress the expression of adjacent genes by altering sequence methylation levels through RNA‐directed DNA methylation (RdDM). In this process, CHH (H = A, C or T) methylation is primarily guided by small‐interfering RNAs (siRNAs) and catalyzed by the DNA methyltransferase domains rearranged methyltransferase‐2 (DRM2) (Cao & Jacobsen, 2002; Calarco *et al*., 2012). Dicer‐like proteins (DCL4, DCL2 and DCL3) generate siRNAs (Axtell, 2013; Wei *et al*., 2014) that are subsequently incorporated into Argonaute (AGO) proteins, particularly AGO3, AGO4, AGO6 and AGO9 specialized for RdDM‐associated siRNAs. The AGO–siRNA complex then mediates the RdDM process, thereby regulating gene expression at multiple levels (Axtell, 2013). Mi‐549 may play a crucial role in regulating the pollen‐specific expression of *Sli* and its strict gamete selection in progeny (Chen *et al*., 2014; Eggers *et al*., 2021; Ma *et al*., 2021). However, the mechanism underlying the Mi‐549‐regulated *Sli* expression remains largely unknown. Furthermore, what is the evolutionary origin of Mi‐549 within the *Solanaceae* family? Is the rise of Mi‐549 accompanied by the SC phenotype in diploid potatoes?

In this study, we revealed that the Mi‐549 is associated with a decreased DNA methylation level within the *Sli* promoter and affects the tissue‐specific expression of *Sli*. In addition, systematic analysis of the whole‐genome sequencing (WGS) data of 393 diploid lines showed that Mi‐549 originated first in wild relatives and is mostly associated with *Sli* expression. More importantly, three new SC diploid lines from *S. lesteri*, *S. neocardenasii* and *S. stenotomum* groups were identified from the screening, which expand the current germplasms pool and hold significant value for hybrid potato breeding.

## Materials and Methods

### Plants and materials

The diploid potato used for the self‐pollination test was sourced from the International Potato Center (Centro Internacional de la PAPA (CIP)). The potato material was propagated from tissue culture seedlings and grown in a solar glasshouse under controlled conditions: daytime temperature of 25°C, night‐time temperature of 18°C, 16 h of light and 70% humidity. *Nicotiana benthamiana* Domin seeds were sown in a nutrient soil–perlite mix, transplanted into pots 10 d after germination and grown under the same environmental conditions as the potato. Detailed information about the planting materials and bioinformatic analyses is provided in Supporting Information Table S1.

### Dual luciferase assay

The 2000 bp *Sli* gene promoter and truncated fragments from RH (with the Mi‐549 insertion) and PI 225689 were cloned into the pGreenII 0800‐LUC reporter vector to drive the expression of the *firefly luciferase* gene (*LUC*). The CaMV 35S promoter‐driven *Renilla (REN) luciferase reporter* gene was used as an internal reference standard. The dual luciferase assay was performed according to the manufacturer's instructions (E1910; Promega). Luciferase activity was measured using Thermo Fisher VARIOSKAN LUC 3 d after injection (Wang *et al*., 2024). Mock with empty vector served as the negative control and 35s promoter‐driven *LUC* was used as the positive control. To ensure robust comparisons, each leaf sample includes positive control, negative control and cross‐experimental group treatments. Before designing the truncation experiments, potential transcription factor (TF)‐binding sites on the *Sli* promoter were predicted using the PlantPAN database (http://plantpan.itps.ncku.edu.tw/plantpan4/index.html), and the *Sli* promoter was truncated based on these predictions (Chow *et al*., 2024). Primer information and data are provided in Tables S2 and S3. The results are presented as the ratio between LUC and REN activity from 14 or 9 independent biological replicates.

### RNA extraction and real‐time‐quantitative polymerase chain reaction (RT‐qPCR) analysis

Total RNA was extracted from plant pollen/anthers and leaf tissues using Vezol reagent (R411‐01; Vazyme, Nanjing, China), following the manufacturer's instructions. One microgram of total RNA was then treated with the HiScript IV RT SuperMax kit for quantitative polymerase chain reaction (qPCR; with gDNA wiper) (R423‐01; Vazyme). Real‐time quantitative polymerase chain reaction analysis was performed using ChamQ SYBR qPCR Master Mix (Q311‐02; Vazyme) to quantify three samples. *Actin* (*PGSC0003DMG400023429*) and *tubulin* (*PGSC0003DMG400009938*) were used as internal reference genes (Tang *et al*., 2017). Real‐time quantitative polymerase chain reaction was carried out on an Applied Biosystems 7500 Real‐Time PCR System (Thermo Fisher Scientific, Waltham, MA, USA ) with SuperReal Fluorescence quantitative premixing reagent (probe method FP206; TIANGEN, Beijing, China) in a 20 μl reaction volume. The *Sli* probe was modified with 5′‐FAM and 3′‐TAMRA‐N. Data were analyzed using the 2^−ΔΔCT^ method (Livak & Schmittgen, 2001), and gene expression data are available in Table S4.

### Bisulfite sequencing and DNA methylation analyses

Genomic DNA was extracted from potato leaves and pollen following the manufacturer's instructions (DP305; TIANGEN). RNA contamination was removed, and the DNA was treated using the EZ DNA methylation‐Gold kit (D5005; Zymo Research, Orange County, California, USA), followed by purification. The treated DNA was then amplified via PCR using ZymoTaq PreMix (E2003; Zymo Research). Polymerase chain reaction products were cloned into the pEASY‐Blunt cloning vector (CB101‐01;TransGen Biotech, Beijing, China), and individual clones were sequenced (Huang *et al*., 2018). Sequencing data were analyzed using Kismet, available at the Kismet website, to calculate the methylation level of each fragment (https://katahdin.girihlet.com/kismeth/revpage.pl) (Gruntman *et al*., 2008). The primers used for bisulfite sequencing are listed in Table S5.

### Identification of the coverage of the Mi‐549 in *Petota* accessions

The *Petota* DNA re‐sequence data were collected from the public platform based on the project ID in the researches. The quality of the Illumina sequencing data was controlled by fastp (Chen *et al*., 2018). Reads of the 393 accessions were mapped to the RH reference genome using BWA ‘aln’ and ‘sampe’ with default parameters (Table S1) (Li & Durbin, 2009; Zhou *et al*., 2020). The mapped reads were sorted, and duplicates were marked using Picard's AddOrReplaceReadGroups and MarkDuplicates functions, respectively. The mapped reads quality values bigger than 10 were retained and used to identify the coverage. First, the reads mapped to chr12_2 of the RH genome were filtered, and the depth was calculated using samtools with the ‘depth’ function. Second, the depth of the 549 bp (from 64 820 556 bp to 64 821 104 bp of chr12_2) was checked and the coverage rate value (CV) in each accession (Li *et al*., 2009). Finally, the three groups used to categorize the degree of coverage were defined through the continuity of the CV. The latitude and longitude information of the diploid potato collection site was obtained using the germplasm resources website (www.genesys‐pgr.org), and the distribution map was drawn by ArcGIS (10.3.1) (Table S6). The distribution of Mi‐549 on PG1011 and PG6241 genomes was identified using pan‐genomic data (Table S7).

### PCR amplification of *Sli* promoter

The PCR conditions were as follows: 98°C for 30 s; 35 cycles of 98°C for 10 s, 52°C for 4 s, 72°C for 10 s, and a final extension at 72°C for 1 min. The PCR products were then cloned into the pEASY‐Blunt cloning vector (CB101‐01; TransGen Biotech). Espript 3.0 was used for sequence alignment analysis (Robert & Gouet, 2014). Primer information can be found in Table S2.

### Self‐pollination and pollen tube elongation observation

For each diploid genotype, more than 15 flowers were self‐pollinated (Table S8). At 55 h postpollination, three pistils were collected from each genotype to assess pollen tube elongation. The pistils were immediately immersed in a 3 : 1 mixture of 95% ethanol and glacial acetic acid. After 24 h, the pistils were transferred to a 5 M NaOH solution for softening for an additional 24 h. The pistils were then washed three to five times with double‐distilled water and incubated in a solution of 0.1 M K_2_HPO_4_ (pH 10) containing 0.005 mg l^−1^ aniline blue fluorescent dye for 12 h. The pistil was placed on a glass slide, followed by the addition of 50% glycerol as the mounting medium. The slide was then covered with another slide, and the specimen was examined under a fluorescence microscope (OLYMPUS U‐HGLGPS BX53F2; Tokyo, Japan) (Ye *et al*., 2018). ImageJ v.1.53c was used to measure the ratio of pollen tube extension length to the total length of style after self‐pollination (Table S9).

### Information on diploid potato SC germplasm resources

Based on reports and statistics from the literature, the potato germplasm resource website (www.cultivariable.com), and the phylogenetic tree of potato evolutionary branches, we reconstructed the phylogenetic tree using the 12 pairs of primers of conserved nuclear orthologs reported by Spooner *et al*. (2018) to map their location in the DM6.1 (DM1‐3516R44) genome. Based on the location information, we identified single nucleotide polymorphisms (SNPs) in the 12 pairs of primers in the corresponding germplasms (Lian *et al*., 2025) and reconstructed the phylogenetic tree. The Molecular Evolutionary Genetics Analysis (MEGA) neighbor‐joining tree was used to construct an evolutionary tree based on the identified SNPs (Tamura *et al*., 2007). The detailed statistics on diploid potato SC can be found in Table S10.

### Comparing the expression level of homologous genes with or without MITE in the promoter region

Ten diploid genomes were selected from the pangenome databases to study the expression variation caused by the MITE insertion in the promoter region (Tang *et al*., 2022). The P‐MITE database of potato was used in our pipeline (Chen *et al*., 2014). The 180 011 MITE were found in the DM reference genome, then we used the 180 011 MITE as query to identify the MITE components in the diploid genome. All the MITEs were aligned to the genomes with ‘blastn ‐query ‐evalue 1e‐5 ‐outfmt 6’. Only the alignment with identity bigger than 98% and the gap < 5 bp were retained in the downstream analysis (Mahram & Herbordt, 2015). After identifying all the locations of the MITE, the gene location was collected from the gene structure files. The JCVI pipelines were used to identify the homologous genes in diploid genomes (Tang *et al*., 2024). The jcvi with ‘jcvi.compara.catalog ortholog ‐‐dbtype prot ‐‐no_strip_names’ were used to find the colinear blocks in the diploid genome with protein sequence and gene location files. The colinear blocks with less than 30 pairs of genes were filtered out, the remaining bigger blocks were used as homologous gene blocks to compare the expression level. Taken together, the genes that have an MITE in the 2000 bp upstream region and have a homologous gene without the MITE were collected as a single insertion type. Genes that lacked MITE insertions in the 2000 bp upstream region for both homologous were classified as the ‘No insertion’ type and served as negative controls. Conversely, genes with MITE insertions in the same region for both homologous genes were classified as ‘Both insertion’ type and served as positive controls (Table S11).

The expression level normalized to transcripts per million (TPM) in different tissues was collected from the pangenome project (Zhou *et al*., 2020; Tang *et al*., 2022). The variation between the homologous genes mentioned above was calculated by subtracting the minimum homologous gene expression value from the maximum and then dividing by the maximum. If the maximum of the pair is zero, the pair was excluded. The boxplot describing the variation was generated using the boxplot function in R.

## Results

### Identification of the regulatory core elements within the *Sli* promoter

**Fig. 1:**
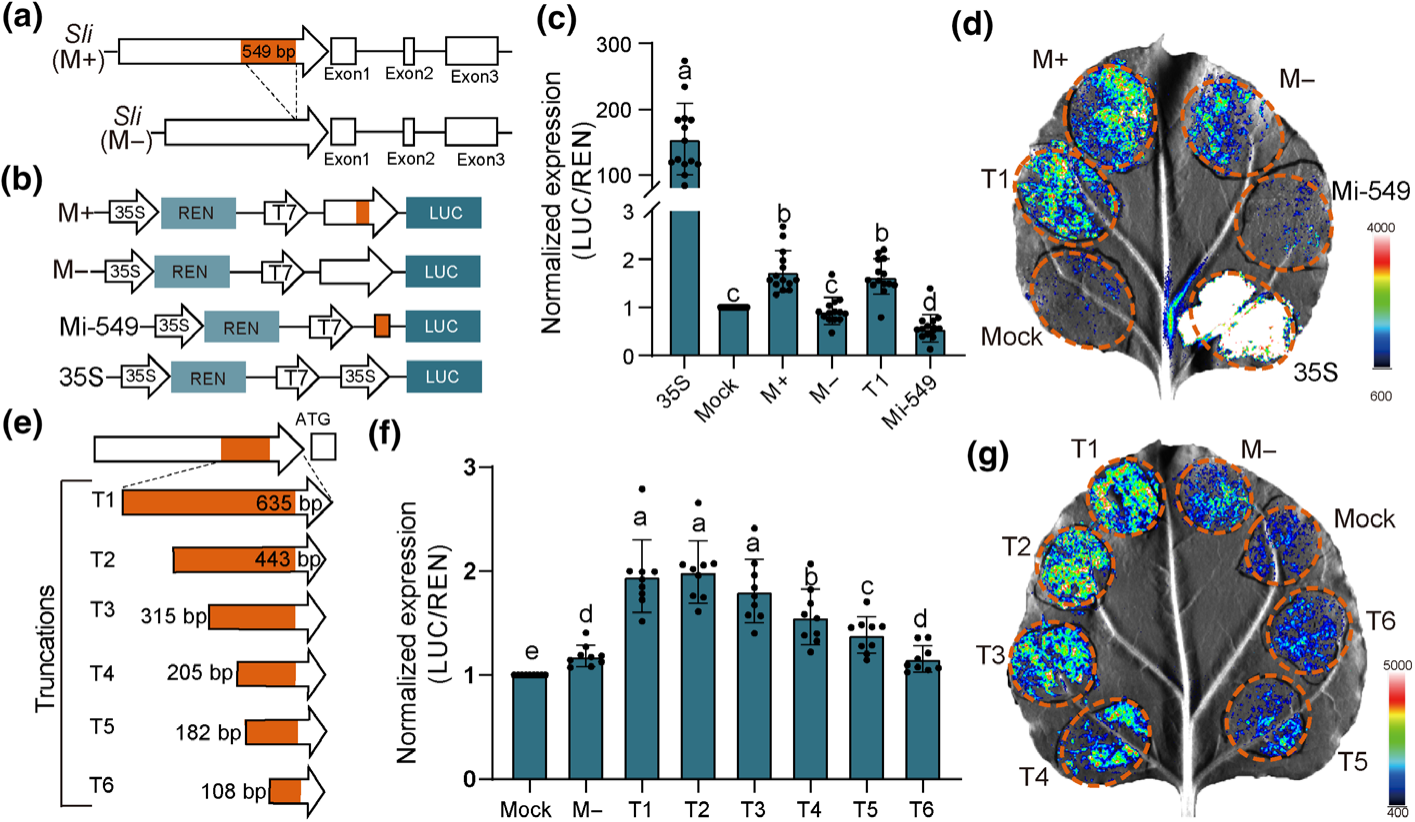
Activity of the *S‐locus inhibitor* (*Sli*) gene promoter. (a) Schematic illustrating the *Sli* gene with (M+) or without (M−) Mi‐549 insertion in the promoter region. (b) Schematic diagrams of various constructs used in the dual luciferase assay. M+ and M− are two types of *Sli* gene promoters with and without Mi‐549. Mi‐549 is the *firefly luciferase* (*LUC*) gene driven solely by Mi‐549. LUC is firefly luciferase; REN is renilla luciferase. The 35S promoter‐driven *LUC* was used as a positive control. (c) Dual luciferase assay showing different activity of the reporter gene on the promoter shown in (b). Data are presented as mean ± SD (*n* = 14). There are significant differences between the different letters (i.e. a, b, c, d) above the bars. *P* < 0.05 in the two‐tailed Student's *t*‐test. (d) Double luciferase detection using M+ and M− of promoters and only Mi‐549 in *Nicotiana benthamiana* leaves. (e) Schematic diagram of truncation of the M+ promoter used in the dual luciferase assay. The *firefly luciferase* gene is driven by Mi‐549 and the core promoter in M+. (f) Dual luciferase assay of the truncated M+ promoter. Data are presented as mean ± SD (*n* = 9). There are significant differences between the different letters (i.e. a, b, etc.) above the bars. *P* < 0.05 in the two‐tailed Student's *t*‐test. (g) Double luciferase detection of the truncated M‐promoter in *Nicotiana benthamiana* leaves.

The *Sli* promoter with Mi‐549 (M+) significantly activates the *Sli* expression in pollen, whereas the promoter without Mi‐549 (M−) cannot (Fig. S1a,b). A similar *Sli* expression pattern was observed in the F_1_ hybrids crossed by PG6068 (M−) and RH (M+) (Fig. S2a–d). To figure out the potential mechanisms underlying *Sli* regulation, detailed activation activity assays were carried out using a dual luciferase system. First, the M+ or M− promoter (Fig. 1a,b) was tested in the dual luciferase assay. As shown in Fig. 1c, the M+ sample exhibits a significantly higher activity than other samples, underscoring the critical role of Mi‐549 in modulating the reporter gene. Notably, Mi‐549 alone cannot confer any activation activity to the downstream gene (Fig. 1b–d), emphasizing the promoter's integrity is indispensable for its activity.

Subsequently, to figure out the core promoter region working with Mi‐549 in activating the reporter gene, the activity of a 635 bp fragment (designated as T1) encompassing Mi‐549 and an 86 bp sequence upstream of the start codon was tested in the assay, which showed a comparable activity to the M+ sample (Fig. 1e–g). This result highlights that T1 contains the core regulatory elements in driving the reporter gene. We then further truncated T1 to 443, 315, 205, 182, and 108 bp, based on potential TF‐binding sites predicted by the PlantPAN database (designated as T2, T3, T4, T5, and T6, respectively, Fig. 1e), and tested their activities. Results showed that the activation capability of T2 and T3 was indistinguishable from that of T1. By contrast, an obvious activity decrease was observed for T4, while a comparable activity to the M− sample was observed for T5 and T6 (Fig. 1f,g). These findings suggested that the sequence fragment (−315 to −182 bp) within Mi‐549 was crucial for gene regulation.

### The potential mechanism underlying the Mi‐549‐regulated *Sli* expression

**Fig. 2:**
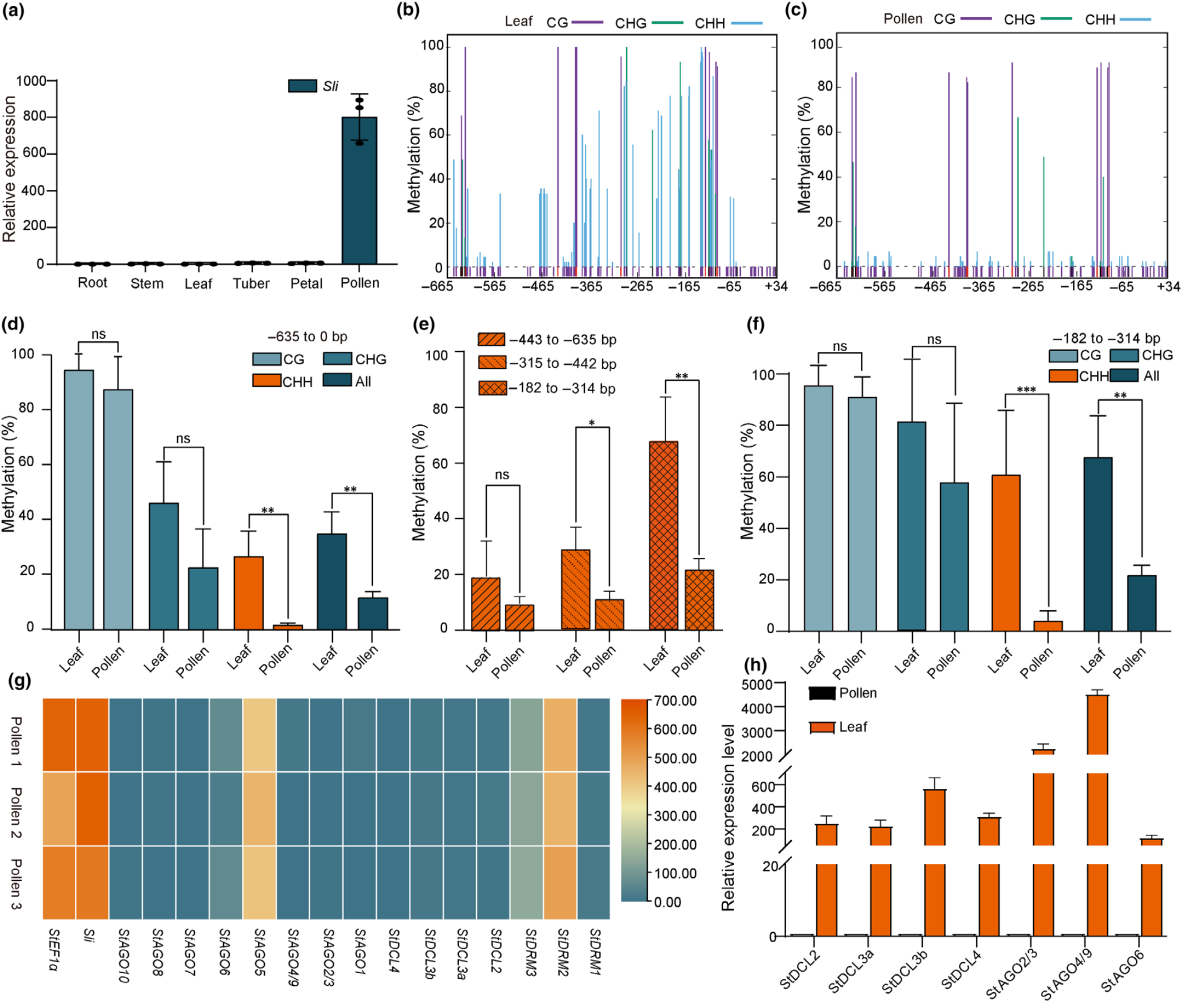
*S‐locus inhibitor* (*Sli*) gene DNA methylation at Mi‐549 region compared between leaves and pollen of RH. (a) Expression pattern of the *Sli* gene in RH. (b, c) Methylation levels in the −665 to +34 bp region of the *Sli* gene promoter in RH: (b) for RH leaf and (c) for RH pollen. Different sequence contexts of the methylation cytosine are denoted by the color of the line. Three independent biological replicates were conducted for each tissue type, with 15 individual clones from each reaction sequenced and analyzed using Kismeth software (Gruntman *et al*., 2008). (d) Cytosine methylation levels within the −635 to 0 bp region, amplified from both RH leaves and pollen, were determined by bisulfite sequencing (BS)‐polymerase chain reaction (PCR). The percentage of methylated cytosines (mC%) among all cytosines in the amplified fragment is indicated. The sequence context of the cytosine is denoted by the color of the bar. Data are presented as mean ± SD (*n* = 3). **, *P* < 0.01 in a two‐tailed Student's *t*‐test. (e) The percentage of mC% in CHH context among all cytosines at different Mi‐549 fragments, amplified from both RH leaves and pollen, was determined by BS‐PCR. Different regions are denoted by the texture of the bar. Data are presented as mean ± SD (*n* = 3). *, *P* < 0.05; **, *P* < 0.01 in a two‐tailed Student's *t*‐test. (f) Cytosine methylation levels within the −315 to −182 bp region of the *Sli* gene promoter in RH leaves and pollen, determined by BS‐PCR. The percentage of methylated cytosines (mC%) among all cytosines in the amplified fragment is indicated. The sequence context of the cytosine is denoted by the color of the bar. (g) Transcriptional profile of genes related to the RNA‐directed DNA methylation (RdDM) pathway in potato pollen. (h) Expression levels of genes related to the RdDM pathway in potato leaves and pollen. Data are presented as mean ± SD (*n* = 3).

A detailed sequence analysis of Mi‐549 reveals that it belongs to the hAT superfamily of MITEs (Fig. S3), which have been found to increase the methylation level of the MITE inserted region to inhibit the expression of adjacent genes (Tan *et al*., 2016; Xu *et al*., 2020; Hu *et al*., 2024). However, in *Arabidopsis* sperm cells, DNA methylation undergoes reprogramming compared to somatic cells, leading to an obvious decrease in the methylation level, especially the CHH methylation, in mature pollen (Slotkin *et al*., 2009; Calarco *et al*., 2012). Since *Sli* is highly and specifically expressed in pollen (Fig. 2a; Table S4), we hypothesize that the reprogramming of DNA methylation levels, distinct from that of somatic cells, may occur in the pollen tissue and trigger the pollen‐specific expression of *Sli*.

To test this hypothesis, we employed bisulfite sequencing (BS)‐PCR to compare the DNA methylation level of T1 between the pollen and leaf of RH (Fig. S4). As shown in Fig. 2b–d, although no statistically significant difference was observed for CG and CHG methylation levels, the CHH methylation is obviously lower in pollen (1.86%) than in the leaf (26.93%), largely accounting for the lower total methylation level observed in the pollen (Fig. 2b–d; Table S5). To clarify the mechanism underlying the differential CHH methylation in pollen, we analyzed the expression of genes associated with the RdDM pathway in mature pollen. RdDM‐related genes in potato were obtained by phylogenetic tree analysis (Fig. S5a,b), and in addition to the *StDRM2* gene, *StDRM3* was also expressed at low levels (Fig. 2g), whereas in *Arabidopsis*, *DRM2* was considered to be a DRM‐like gene that functions in the pollen vegetative nucleus (Cao & Jacobsen, 2002; Calarco *et al*., 2012). Consistent with the findings in other plant species (Grant‐Downton *et al*., 2009), the expression of *StDCL3*, which generates 23/24nt siRNAs, was not observed in the mature potato pollen (Fig. 2g,h). In addition, among *AGO* genes, only *StAGO5* was expressed, whereas AGOs expressing RdDM‐related siRNAs were detected in leaves (Fig. 2g) (Axtell, 2013; Deng *et al*., 2017). These findings suggest that the absence of *StDCL3‐* and RdDM‐associated *StAGO* genes in mature pollen likely contributes to the loss of CHH methylation in the pollen (Fig. S5c).

Subsequently, to investigate the relationship between the activity and the methylation level of the core regulatory element within the *Sli* promoter, we compared the methylation levels of the truncated promoters and found that the most significant methylation level difference between potato pollen (4.2%) and leaf (60.8%) occurred at the core fragment (−315 to −182 bp within Mi‐549, Figs 1e,f, 2e,f). Furthermore, we also analyzed the methylation levels of M+ (Mi‐549) transiently expressed in tobacco leaves used in the dual luciferase reporter system. The results revealed that Mi‐549 exhibited low methylation levels in tobacco leaves (Fig. S6), suggesting that Mi‐549 is not subject to extensive methylation modification in tobacco leaves, which is consistent with its high transcriptional activity in tobacco leaves. Since the decreased CHH methylation within MITE can enhance the expression of adjacent genes in citrus (*Fortunella hindsii*) and rice (*Oryza sativa*) (Deng *et al*., 2017; Huang *et al*., 2018; Xu *et al*., 2020), Mi‐549 can regulate the pollen‐specific expression of *Sli* in a similar way.

### Evolutionary origin and potential impact of Mi‐549 in *Solanum*

**Fig. 3:**
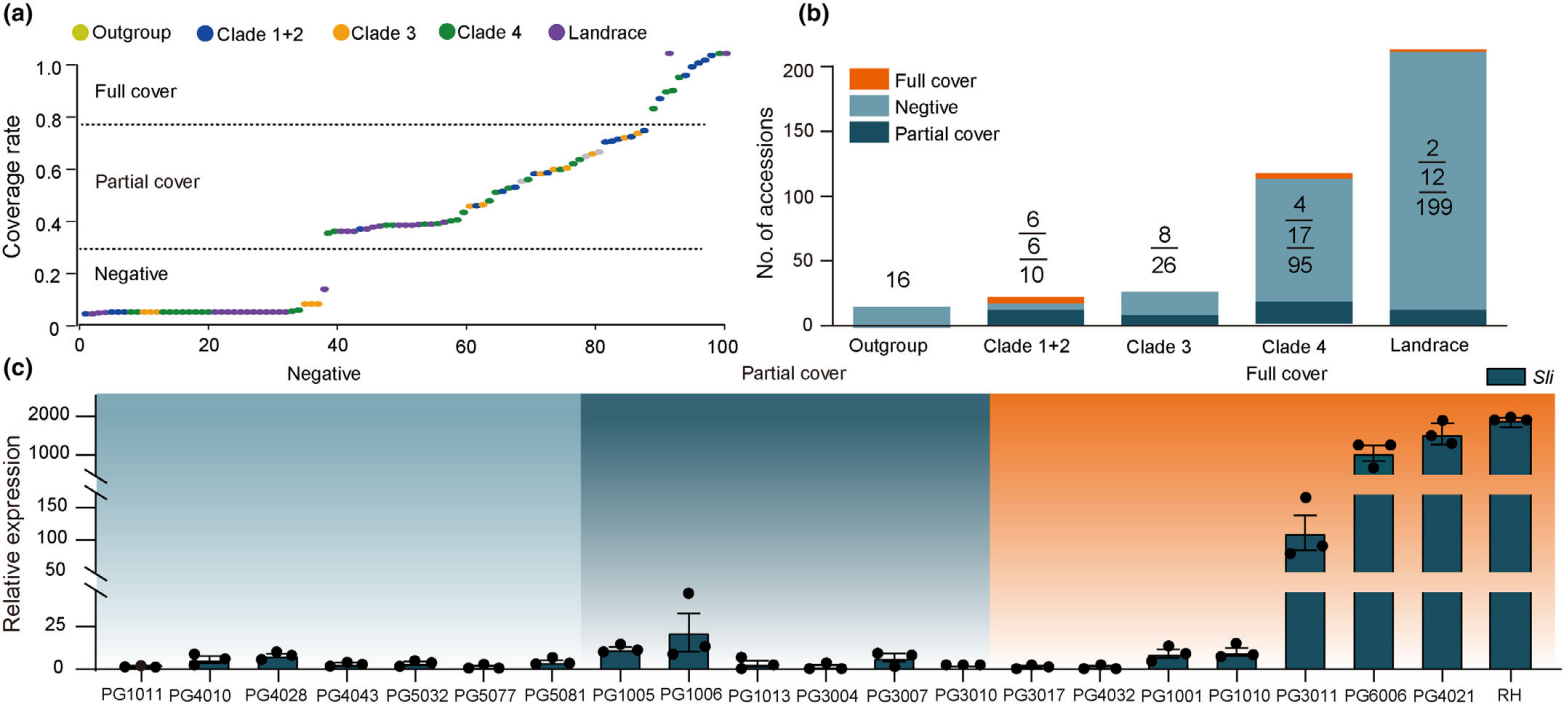
Distribution of Mi‐549 in *Solanum* and its impact on the expression pattern of the *S‐locus inhibitor* (*Sli*) gene. (a) Coverage rate value of corresponding region in 393 whole‐genome sequencing datasets over Mi‐549 region (from 64 820 556 bp to 64 821 104 bp of the chr12_2) in RH. The dashed lines indicated the thresholds used for classing the 393 genotypes used in coverage rate analysis into three groups: negative (below 0.3), partial cover (from 0.3 to 0.75), l cover (above 0.75). (b) The numbers of three coverage types (classed in a) in different groups categorized by Spooner's classification. The numbers on the bar indicate the number of accessions at the corresponding positions. The coverage types are denoted by the color of the bar. (c) Expression levels of *Sli* gene in anthers of genotypes with different Mi‐549 insertion types by quantitative real‐time polymerase chain reaction. Data are presented as mean ± SD (*n* = 3).

Subsequently, to comprehensively assess the origin and potential impact of Mi‐549 in *Solanum*, the WGS data of 393 diploid lines, categorized into outgroup (*n* = 16), clades 1 + 2 (*n* = 22), clade 3 (*n* = 26), clade 4 (*n* = 116), and landrace (*n* = 213) (Spooner *et al*., 2014), were used in the analysis. Mi‐549 is located on chromosome 12_2, spanning from 64 820 556 to 64 821 104 bp in the RH genome (Zhou *et al*., 2020). The 393 WGS data sets were then aligned to the position of Mi‐549, and the CV was calculated to catalog the 393 accessions into three catalogs: negative (CV < 0.3, *n* = 333), partial coverage (CV = 0.3–0.75, *n* = 47), and full coverage (CV > 0.75, *n* = 13) (Fig. 3a; Table S6). Among these accessions, the 16 nontuberizing outgroups, including *S. etuberosum*, *S. palustre*, and *S. fernandezianum*, do not have Mi‐549, while clade 1 + clade 2 has a significantly higher proportion of full Mi‐549 (27.3%, *n* = 6) and clade 3 only has partial Mi‐549 (30.8%, *n* = 8) (Fig. 3b). This analysis indicates that Mi‐549 may initially undergo random insertion into the *Sli* promoter region in PG1010 of *S. lesteri* (Spooner *et al*., 2018; Tang *et al*., 2022) and subsequently spread to other *Petota* species through a sexual event (Fig. S7; Table S7). In clade 4, the proportion of full and partial Mi‐549 was 4.3% (*n* = 4) and 13.8% (*n* = 17), respectively. Among the landrace accessions, a similar low CV is observed for full (0.9%, *n* = 2) and partial Mi‐549 (5.6%, *n* = 12). Since potatoes are primarily propagated asexually through tubers, there is minimal selection pressure to maintain fertility traits that favor SC. This may explain why recent populations, such as clade 4 and cultivated landraces, have fewer lines with Mi‐549.

To further investigate the impact of Mi‐549 on *Sli* expression in different genetic backgrounds, we collected the anthers of the representative lines from full, partial, or no Mi‐549 coverage groups (Fig. 3a,b) and measured the expression level of the *Sli* gene in anthers of these lines (Table S4). As depicted in Fig. 3(c), the diploid lines with full Mi‐549 exhibit a higher *Sli* expression than those with partial or no Mi‐549, although notable exceptions were also observed (e.g. PG3017, PG4032, PG1001, PG1010), indicating other unknown factors may also participate in the *Sli* regulation.

### Identification of three new SC lines from the diploid germplasm

**Fig. 4:**
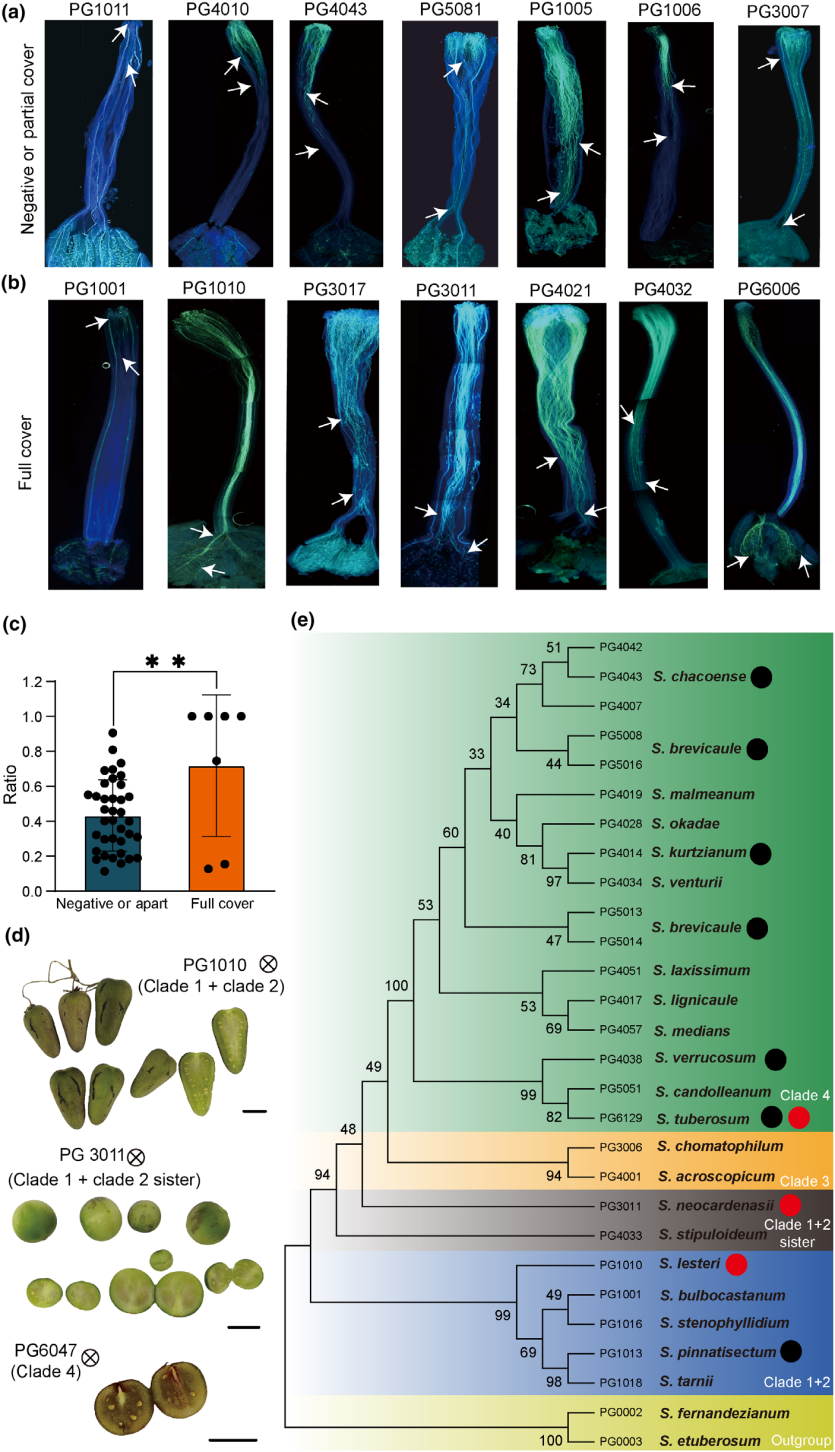
Impact of Mi‐549 insertion on pollen tube elongation after self‐pollination and screening of self‐compatibility (SC) *Petota*. (a, b) Pollen tube growth in self‐pollinated styles in diploid potatoes without or with partial Mi‐549 insertion (a) and with full Mi‐549 insertions (b) after 55 h of self‐pollination. White arrows indicate the termination point of the pollen tube. (c) Proportion of pollen tubes that reached most of the style length after germination. Significance analysis was performed by one‐tailed Student's *t*‐test: **, *P* < 0.01. (d) Accessions in which the pollen tube successfully reached the ovule after self‐pollination and set fruit normally. (e) The diploid potato SC accessions are positioned on the species tree as follows: Black dots represent potato species with SC accessions reported in previous studies, whereas red dots refer to the SC accessions identified in this study using the Mi‐549 marker (De Jong & Rowe, 1971; Olsder & Hermsen, 1976; Jansky *et al*., 2014; Peterson *et al*., 2016; Fulladolsa *et al*., 2019; Zhang *et al*., 2019a,b; van Lieshout *et al*., 2020; Yang *et al*., 2020; Kaiser *et al*., 2021; Ames *et al*., 2024).

In search of the evolutionary origin of Mi‐549, we examine the SC/SI phenotype of *Petota* (Fig. S8), which is essential for hybrid potato breeding. We initially selected 14 accessions, including 7 with no or partial Mi‐549 and 7 with full Mi‐549 (Fig. S9). As illustrated in Fig. 4(a,b), the lines with full Mi‐549 (lower panel) exhibited a significantly higher rate of pollen tube elongation than lines with no or partial Mi‐549 (upper panel) *c*. 55 h after self‐pollination. We then expanded the survey to 44 accessions consisting of 37 with no or partial Mi‐549 and 7 with full Mi‐549 (Fig. S8; Table S3). As revealed by a statistical result, similar results were observed: The pollen tube elongation rate was significantly higher in the accessions with full Mi‐549 than those with no or partial Mi‐549 (Fig. 4c, S8; Table S9). Ultimately, we found four accessions (PG1010, PG3011, PG6006, PG6047), whose pollen tubes could successfully reach the ovary after selfing.

More importantly, PG1010 and PG3011 successfully produced fruits after self‐pollination, whereas PG6047 produced only one fruit (*n* = 3 pollinations) (Fig. 4d), and PG6006 failed to produce fruit (*n* = 2 pollinations; Fig. S10) likely due to flower bud abortion (Fig. S11). As most of the currently reported SC diploid potatoes originate from *S. chacoense* and *S*. *tuberosum* Group *stenotomum* (Table S10) (De Jong & Rowe, 1971; Olsder & Hermsen, 1976; Jansky *et al*., 2014; Peterson *et al*., 2016; Fulladolsa *et al*., 2019; Zhang *et al*., 2019a,b; van Lieshout *et al*., 2020; Yang *et al*., 2020; Kaiser *et al*., 2021; Ames *et al*., 2024), the newly identified SC lines derive from *S. lesteri* and *S. neocardenasii*, both of which belong to clade 1 + 2 and sister branch, respectively (Fig. 4e) (Spooner *et al*., 2018), providing a new gene resource primarily associated with stress response (Bozan *et al*., 2023), and strong resistance against pests such as *Globodera pallida*, *Leptinotarsa decemlineata*, and *Myzus persicae* (Wright *et al*., 1985; Lapointe & Tingey, 1986; Castelli *et al*., 2003). Thus, these SC lines would expand the current germplasm pool and hold significant value for breeding applications.

### Whole‐genome transcription affected by MITEs in *Petota*

**Fig. 5:**
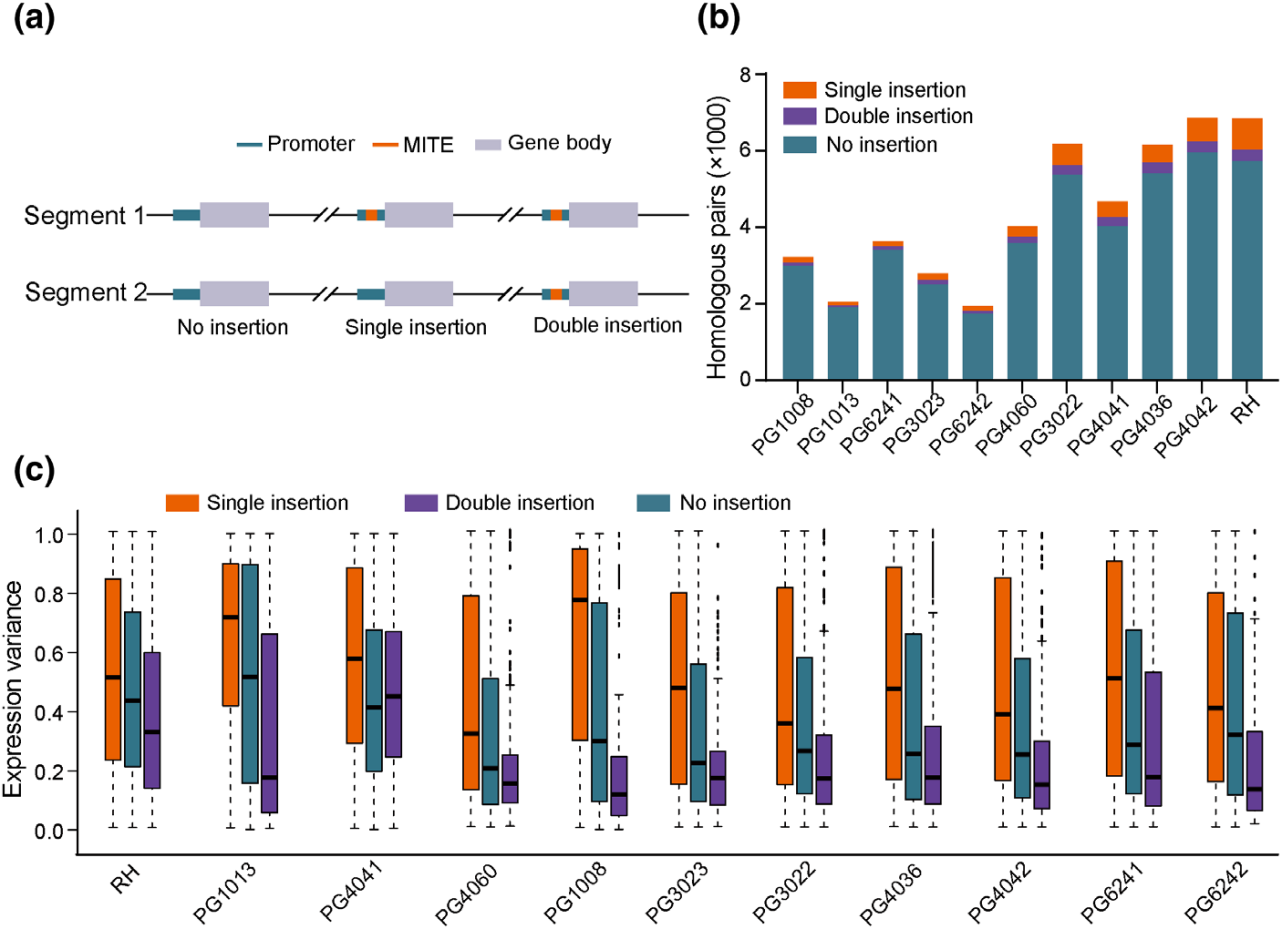
Miniature inverted‐repeat transposable element (MITE) insertions verify homologous gene pair expression levels in different germplasms. (a) Schematic diagram of the analysis of gene expression variation affected by MITEs. MITE insertions in the promoter regions of homologous gene pairs in the potato genome. (b) Histogram of the count of homologous pair MITE insertion types in 11 germplasms. (c) Boxplot of the expression variation between the homologous gene pairs with different MITE insertion types. Single insertion, only one gene of the homologous pair contains a MITE in the promoter region (2 kb in the upstream genes). No insertion, no gene of the homologous pair has an MITE in the promoter region. Double insertion, both genes of the homologous pair have MITE in the promoter region. The upper and lower boundaries of the colored box represent the third quartile (Q3) and the first quartile (Q1), respectively. The horizontal line in the middle of the colored box indicates the median. The dashed lines extending beyond the box reach to the vertical lines that mark the minimum and maximum values within 1.5 times the interquartile range (IQR = Q3–Q1) from the quartiles; specifically, the upper limit is Q3 + 1.5 × IQR and the lower limit are Q1–1.5 × IQR. Data points that fall outside these limits are considered outliers and are indicated as such.

Since the Mi‐549 in the *Sli* promoter plays an essential role in *Sli* regulation, how about the potential roles of other MITEs in the regulation of nearby genes in *Petota*? A total of 387,063 MITEs are annotated in the RH genome based on P‐MITE databases (Chen *et al*., 2014), 6681 of which are located within the promoter region (upstream 2000 bp from the start codon) of coding genes. Given that kallisto's pseudoalignment of RNA‐seq reads to a reference genome can accurately quantify the expression level in a homologous‐specific manner (Bray *et al*., 2016), this method could be used to quantify the expression differences between homologous genes having MITE or no MITE in their promoters (Fig. 5a). To ensure the accuracy of this analysis, only syntenic blocks containing at least 30 homologous gene pairs were included in the comparison, which identified a total of 6853 homoeologous gene pairs.

Among 830 gene pairs, only one gene promoter contains MITE and the other has no MITE, whereas 290 gene pairs, used as negative controls, contain MITE in both gene promoters (Fig. 5b, Table S12). Subsequently, the expression patterns of 1120 homologous pairs (830 + 290) were analyzed. Consistently, when one promoter has the MITE and the other has no insertion (*n* = 830), the greatest gene expression difference was observed between homoeologous genes. By contrast, when both promoters have MITEs (*n* = 290), only small expression variance was observed for the gene pairs, probably due to a similar regulation conferred by the MITE to the homologous genes (Fig. 5c). Furthermore, we notice that this gene expression variation affected by the MITE is not sensitive to tissue differences such as root, stem, leaf, stolon, and tuber (Fig. S12).

We further applied qRT‐PCR analysis to confirm the results revealed by the RNA‐seq data, and similar patterns were observed (Fig. S13), indicating MITEs play an essential role in regulating the expression of nearby genes in RH. To examine whether the gene expression pattern is universally affected by MITE in potato, we used another 10 diploids selected based on phylogenetic relationships (Fig. 5b) (Chen *et al*., 2014; Tang *et al*., 2022), and similar regulatory effects were observed for the MITEs (Fig. 5c, S14). Therefore, we conclude that MITEs are essential regulatory elements in controlling gene expression in diploid potatoes.

## Discussion

*Sli* is a ‘master key’ gene capable of conferring SC to most of the SI diploid lines and thus plays an essential role in hybrid potato breeding (Eggers *et al*., 2021; Ma *et al*., 2021). In this study, we showed that the MITE (Mi‐549) in the *Sli* promoter is an indispensable core element in regulating the pollen‐specific expression of *Sli* (Fig. 1c,f). Interestingly, according to our results (Fig. 2b–d) and other studies (Xu *et al*., 2020; Hu *et al*., 2024), MITEs usually increase the sequence methylation level at the insertion, which in turn inhibits the expressions of the nearby genes. However, for Mi‐549, instead of enhancing methylation, its methylation level, especially of CHH methylation, decreases in pollen. The loss of CHH methylation within the MITE was also observed in the promoter of the *PigmS* gene in rice, due to a reprogramming of DNA methylation in sperm cells, and thus leads to a specific *PigmS* expression pattern in pollen (Slotkin *et al*., 2009; Deng *et al*., 2017). Studies in *Arabidopsis thaliana* have demonstrated that the loss of *DRM2* expression in sperm cells and the absence of 24 nt siRNA biogenesis genes *DCL3* in mature pollen are major contributors to DNA methylation reprogramming during pollen development (Cao & Jacobsen, 2002; Calarco *et al*., 2012). By analyzing the expression of related genes in potato pollen, we found that the absence of the expression of RdDM‐associated *StDCL* and *StAGO* genes in mature pollen likely contributes to the loss of CHH methylation in pollen (Fig. 2g,h), indicating a similar mechanism should be involved in the regulation of *Sli* expression in RH (Fig. S5).

In addition to Mi‐549, results from both the activity activation assay (Fig. 1c,d) and the wild relatives having Mi‐549 but low *Sli* expression (Fig. 3c) suggest that there are other unknown factors involved in *Sli* regulation. For instance, a TF *FhARI* was reported to bind to the MITE in the *RWP* promoter to control gene expression and apomictic reproduction in citrus (Wang *et al*., 2022). Thus, there may be certain tissue‐specific TFs that can be recruited by the low CHH methylation region within Mi‐549 or the 86 bp sequence upstream of the start codon to activate *Sli* transcription in pollen, which need future investigations.

Given the significance of Mi‐549 in regulating *Sli* expression, we aimed to trace the origin and distribution of Mi‐549 within Solanaceae. In a previous study, using the 133 individuals from 22 wild species relatives of potato and eight diverse cultivated potato clones, we have shown that the accessions from *S. brevicaule*, *S. tuberosum*, *S. chacoense*, and *S. hougasii* harbor the Mi‐549 (Kardile *et al*., 2022; Ames *et al*., 2024). Our analysis of WGS data from 393 diploid lines showed that the Mi‐549 is unique to the *Solanum* section *Petota*, with its earliest occurrence identified in PG1010 of *S. lesteri*, which belongs to clade 1 + 2 (Fig. S7).

Moreover, consistent with Ames's findings, a low frequency of Mi‐549 was observed in clade 4 and the landrace accessions (Fig. 3a,b), indicating Mi‐549 is not selected during domestication. In rice, MITEs can enhance plant adaptability to cold and salt stress by regulating the transcription of nearby genes (Naito *et al*., 2009), and thus TEs are significantly enriched in selection regions (Li *et al*., 2024). By contrast, potatoes are primarily propagated asexually, which results in low selection pressure for fertility‐related traits (Bozan *et al*., 2023). Additionally, severe inbreeding depression is observed in the selfed progeny of germplasm carrying *Sli*, such as increased rates of bud abortion, absence of flower bud formation, and sterility (Hosaka, 2000). Thus, it is plausible that high expression of *Sli* activated by the Mi‐549 to confer SC diploid potatoes has been minimally selected during evolution (Fig. 3a,b; Table S1).

Although SC trait is not favored during domestication, the identification of SC accessions from *Petota* is still one of the major challenges for the successful production of inbred lines (Gavrilenko *et al*., 2013; Bozan *et al*., 2023). In this study, we identified two SC lines belonging to clade1 + 2 and the sister branch, providing a new gene resource primarily associated with stress response (Bozan *et al*., 2023) and pest resistance (Wright *et al*., 1985; Lapointe & Tingey, 1986; Castelli *et al*., 2003). The three newly identified SC diploid accessions provide considerable promise for hybrid breeding, offering their potential use in developing inbred lines, serving as donors of the SC gene, and possibly introducing valuable resistance genes.

Interestingly, we also observed a high level of *Sli* expression in the anthers of PG4021, but this line exhibits SI after self‐pollination. One possible reason for this unexpected observation is that Sli protein may fail to interact with the *S*‐RNases of PG4021 to degrade these toxins since a high degree of *S*‐RNase polymorphism with amino acid similarity ranging from 32.9% to 94.5% was observed in *Solanum* species (Dzidzienyo *et al*., 2016). In addition, other pistil‐modifying factors such as *HT‐B* and *Trxh* may also contribute to SI besides *S*‐locus regulation.

In summary, our study uncovers the mechanism underlying the Mi‐549‐mediated regulation of the pollen‐specific expression of *Sli*, and the widespread impacts of MITEs on the nearby genes in diploid potatoes. Although Mi‐549 and its impacts on *Sli* as well as SC seem not to be favored during selection, the SC plants with distinct genetic backgrounds are crucial for hybrid potato breeding. Thus, the newly identified SC lines would greatly expand the current germplasm pool and hold significant value for breeding applications.

## Competing interests

None declared.

## Author contributions

YS, LM and QL conceived and organized the study. QL and YJ performed bioinformatics analysis. SZ, XZ and ZZ performed self‐pollination phenotypic analysis. SZ and ZZ performed promoter activation activity assays and methylation level assays. XZ performed fluorescence quantitative data detection. SZ and QL wrote the manuscript. YS and LM revised the manuscript. SZ, QL and ZZ contributed equally to this work.

## Disclaimer

The New Phytologist Foundation remains neutral with regard to jurisdictional claims in maps and in any institutional affiliations.

## Supporting information


**Fig. S1** The relationship between Mi‐549 and the expression pattern of the *Sli* gene.
**Fig. S2** The M+ promoter enhances *Sli* gene expression and influences SC.
**Fig. S3** Sequence alignment of the 549 bp insertion with an hAT‐like transposon from the P‐MITE database.
**Fig. S4** Cytosine methylation levels of the Mi‐549 region and −86 bp.
**Fig. S5** RdDM‐related genes in potato were obtained by phylogenetic tree analysis and CHH methylation patterns in pollen.
**Fig. S6** Methylation level of Mi‐549 in tobacco leaves.
**Fig. S7** Geographic distribution of germplasm with partial and complete Mi‐549 insertions across the Americas.
**Fig. S8** Pollen tube elongation in the 44 diploid potato accessions following self‐pollination.
**Fig. S9** Alignment of *Sli* promoter DNA sequence between the 13 accessions with full Mi‐549 based on WGS data.
**Fig. S10** Comparison of *Sli* promoter DNA sequences between PG6006 and RH.
**Fig. S11** The inbreeding depression gene *FBA1* affects abnormal flower development in PG6047 and PG6006.
**Fig. S12** Effect of MITE insertion in the promoter region on the expression variation of homologous genes in RH.
**Fig. S13** Validation of the MITE insertion induced homologous gene expression difference using RNA‐seq and qRT‐PCR.
**Fig. S14** Effect of MITE insertion in the promoter region on homologous gene expression variation within the diploid potato genome.

**Table S1** List of 393 Solanaceae accessions used to identify the *Sli* promoter Mi‐549 coverage rate.
**Table S2** Primers used in this study.
**Table S3** Tobacco dual luciferase assay results.
**Table S4** Fluorescence quantitative expression data.
**Table S5** Methylation levels of the Mi‐549 and −86 bp regions between leaves and pollen and LUC in tobacco leaves.
**Table S6** List of the 63 accessions with full or partial Mi‐549 coverage.
**Table S7** List of the Mi‐549 homologous in PG1011 and PG6241.
**Table S8** SC/SI phylotype of the 44 diploid accessions with full or partial Mi‐549 coverage.
**Table S9** List of pollen tube length to total style length ratio after self‐pollination of the 44 diploid accessions.
**Table S10** Detailed information of the 27 SC germplasm resources.
**Table S11** SNPs in 12 pairs of nuclear orthologs in diploid potato germplasm resources.
**Table S12** List of MITE insertion in the promoter region of homologous gene pairs in the selected diploid genomes.
Please note: Wiley is not responsible for the content or functionality of any Supporting Information supplied by the authors. Any queries (other than missing material) should be directed to the *New Phytologist* Central Office.

## Data Availability

All data supporting the findings of this study are available in the article or in Supplementary Information files. The RNA‐seq data utilized in this study were obtained from the Chinese National Genomics Data Center (https://bigd.big.ac.cn/) under accession no. CRA002005 as well as the Genome Sequence Archive (GSA) at the National Genomics Data Center (NGDC) (https://ngdc.cncb.ac.cn/gsa/), with BioProject accession no. PRJCA006011. The raw sequencing data reported in this paper have been deposited in the Sequence Read Archive (SRA) under accession no. PRJNA766763 and are publicly accessible. The RNA‐seq data of pollen RdDM pathway genes have been uploaded to the database accession PRJNA1219828. Accession nos. for genes used in Fig. S5: NtDRM1 (BAC67060.1), OsDRM2 (Q10SU5.1), Zmet3 (AF242320), DRM1 (ATF8M21), DRM2 (AF240695), Dnmt3a (AF068625), Dnmt3b (AF068628), Dnmt1 (P13864), DIM‐2 (AF348971), MET1 (P34881), CMT1 (AF039372), CMT3 (AL021711), StDRM1 (Soltu.DM.04G000230.2), StDRM2 (Soltu.DM.02G006560.1) and StDRM3 (Soltu.DM.10G030090.1), Dicer‐1 (NM_079729), Dicer‐2 (NM_079054), hDicer (NM_177438), mDicer (NM_148948), DCL2 (AT3G03300.1), DCL3 (AT3G43920.1), DCL4 (AT5G20320.1), OsDCL3a (Q5N870.1), OsDCL3b (Q7XD96.2), StDCL2 (Soltu.DM.06G011550.1), StDCL3a (Soltu.DM.08G015780.1), StDCL3b (Soltu.DM.08G015790.1), and StDCL4 (Soltu.DM.07G000050.2).
