# Supplementary material for: Origin of a self‐compatibility associated MITE in Petota and its application in hybrid potato breeding
Source: New Phytol. 2025 Mar 31;246(4):1647–59. doi: 10.1111/nph.70093 (PMC12018782; doi:10.1111/nph.70093)
Supplement: Supplementary file 1 — Fig. S1 The relationship between Mi‐549 and the expression pattern of the Sli gene. Fig. S2 The M+ promoter enhances Sli gene expression and influences SC. Fig. S3 Sequence alignment of the 549 bp insertion with an hAT‐like transposon from the P‐MITE database. Fig. S4 Cytosine methylation levels of the Mi‐549 region and −86 bp. Fig. S5 RdDM‐related genes in potato were obtained by phylogenetic tree analysis and CHH methylation patterns in pollen. Fig. S6 Methylation level of Mi‐549 in tobacco leaves. Fig. S7 Geographic distribution of germplasm with partial and complete Mi‐549 insertions across the Americas. Fig. S8 Pollen tube elongation in the 44 diploid potato accessions following self‐pollination. Fig. S9 Alignment of Sli promoter DNA sequence between the 13 accessions with full Mi‐549 based on WGS data. Fig. S10 Comparison of Sli promoter DNA sequences between PG6006 and RH. Fig. S11 The inbreeding depression gene FBA1 affects abnormal flower development in PG6047 and PG6006. Fig. S12 Effect of MITE insertion in the promoter region on the expression variation of homologous genes in RH. Fig. S13 Validation of the MITE insertion induced homologous gene expression difference using RNA‐seq and qRT‐PCR. Fig. S14 Effect of MITE insertion in the promoter region on homologous gene expression variation within the diploid potato genome. [file NPH-246-1647-s002.pdf]

***New Phytologist* Supporting Information**

Article title: Origin of a self-compatibility associated MITE in *Petota* and its application in hybrid potato breeding

Authors: Saihang Zhang, Qinggang Liao, Zhan Zhang, Xu Zhu, Yuxin Jia, Yi Shang, Ling Ma

Article acceptance date: 28 February 2025

The following Supporting Information is available for this article:

Fig. S1 The relationship between Mi-549 and the expression pattern of *Sli* gene.

Fig. S2 The M+ promoter enhances *Sli* gene expression and influences SC.

Fig. S3 Sequence alignment of the 549 bp insertion with a hAT-like transposon from the P-MITE database.

Fig. S4 Cytosine methylation levels of Mi-549 region and -86 bp.

Fig. S5 RdDM related genes in potato was obtained by phylogenetic tree analysis and CHH methylation pattern in pollen.

Fig. S6 Methylation level of Mi-549 in tobacco leaves.

Fig. S7 Geographic distribution of germplasm with partial and complete Mi-549 insertions across the Americas.

Fig. S8 Pollen tube elongation in the 44 diploid potato accessions following self-pollination.

Fig. S9 Alignment of *Sli* promoter DNA sequence between the 13 accessions with full Mi-549 based on WGS data.

Fig. S10 Comparison of *Sli* promoter DNA sequences between PG6006 and RH.

Fig. S11 The inbreeding depression gene *FBA1* affects abnormal flower development in PG6047 and PG6006

Fig. S12 Effect of MITE insertion in the promoter region on the expression variation of homologous genes in RH.

Fig. S13 Validation of the MITE insertion induced homologous gens expression difference using RNA-seq and qRT-PCR.

Fig. S14 Effect of MITE insertion in the promoter region on homologous genes expression variation within the diploid potato genome.

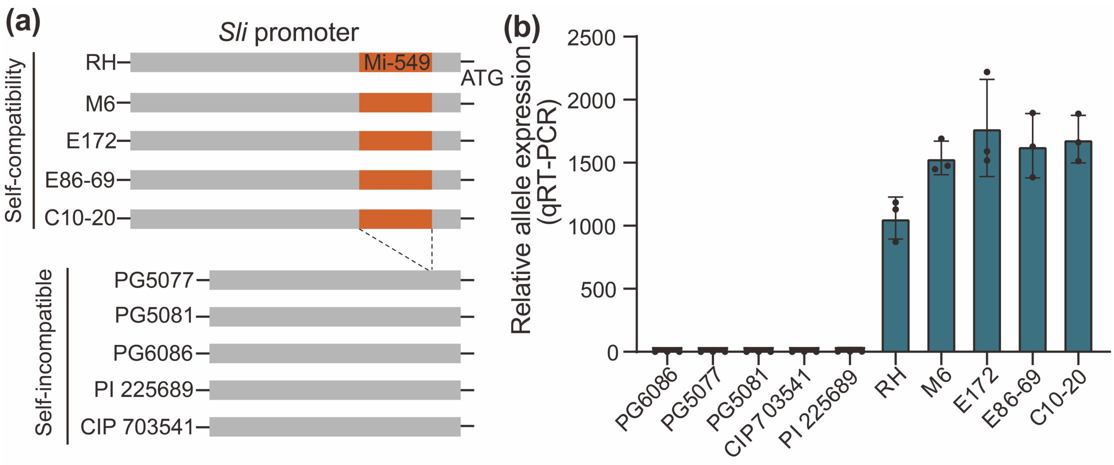

Fig. S1 The relationship between Mi-549 and the expression pattern of *Sli* gene. (a) MITE insertion in the promoter region of the *Sli* gene in diploid SC and SI potatoes. (b) Expression analysis of the *Sli* gene in anthers of SC and SI diploid germplasm.

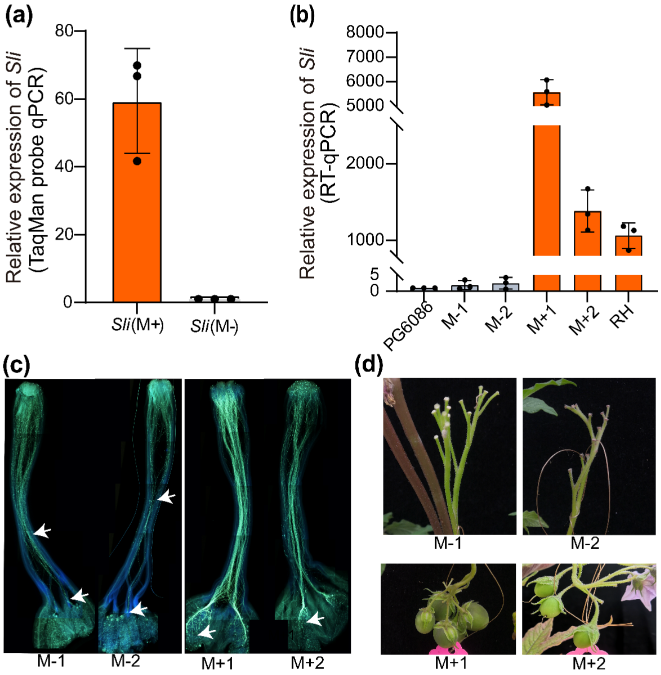

Fig. S2 The M+ promoter enhances *Sli* gene expression and influences SC. (a) Relative expression of the *Sli* gene in RH anthers was detectedusing the probe method. (b) Comparison of *Sli* gene expression in anthers of M+ and M− genotypes in the F1 hybrid of PG6086 and RH. (c) Pollen tube growth in the styles of M+ and M− genotypes following self-pollination in the F1 hybrid of PG6086 and RH. (d) Compatibility of M+ and M− genotypes after self-pollination in the F1 hybrid of PG6086 and RH.

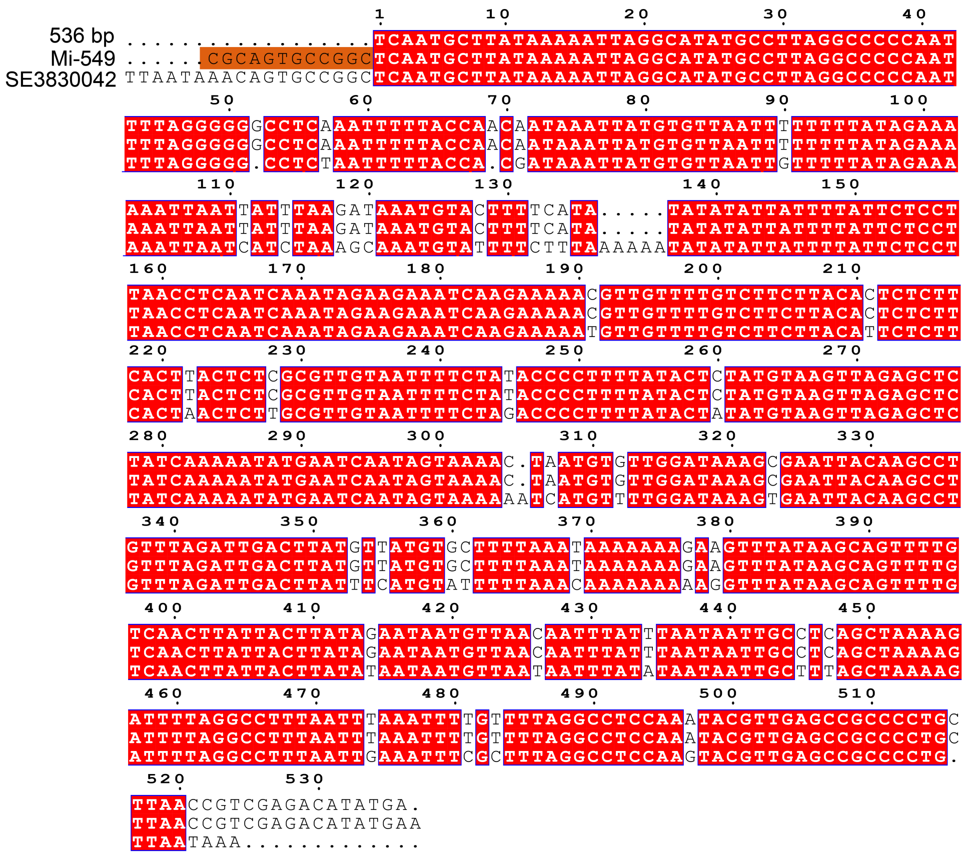

Fig. S3 Sequence alignment of the 549 bp insertion with a *hAT-like* transposon from the P-MITE database.

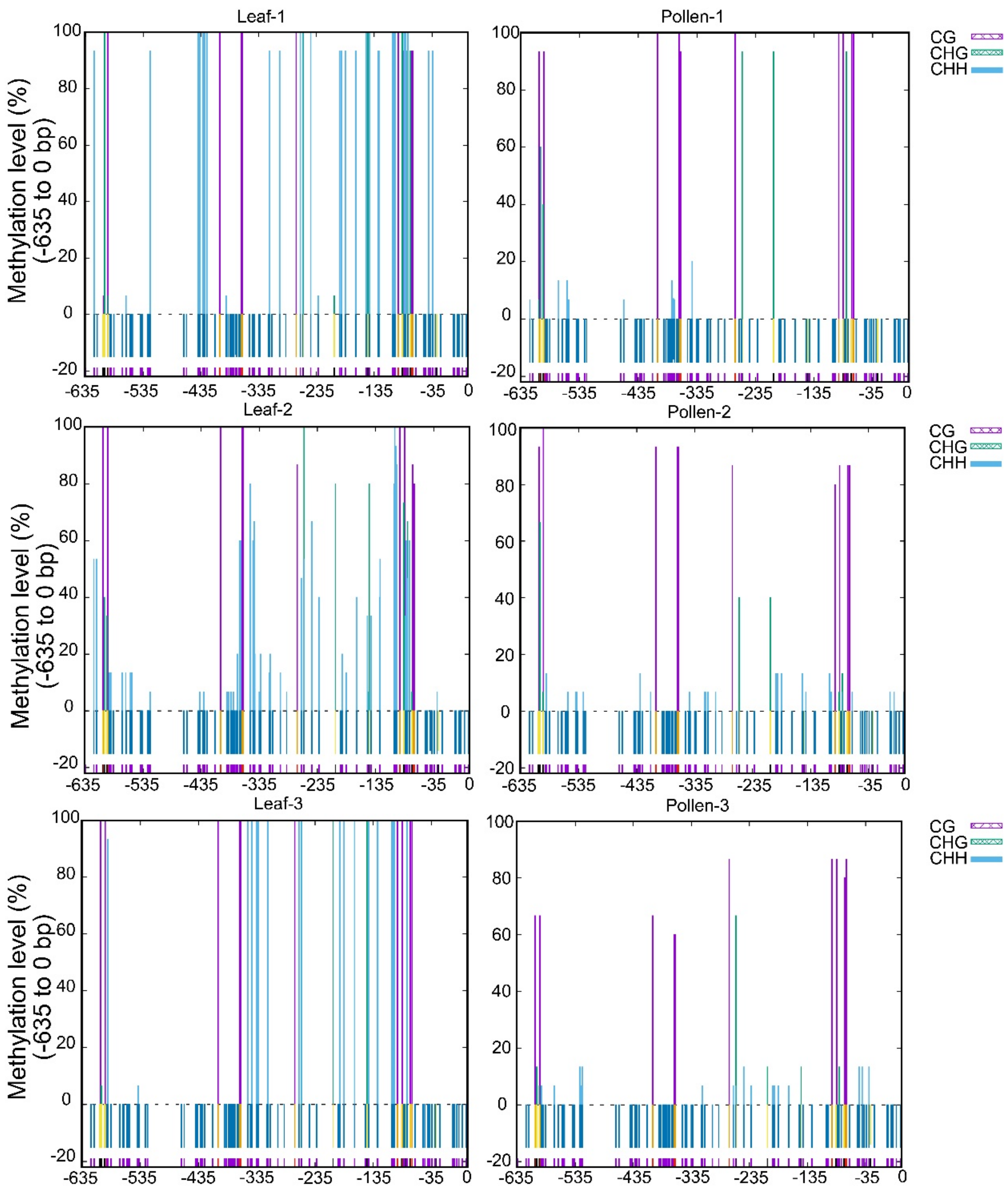

Fig. S4 Cytosine methylation levels of Mi-549 region and -86 bp. a, b Cytosine methylation levels in the Mi-549 and -86 bp region and core promoter in RH. a: Leaf, b: Pollen. The Bisulfite Analysis results were analyzed using the program provided by Ravi LAB (https://katahdin.girihlet.com/kismeth/revpage.pl). The experimental procedure involved three independent biological replicates, with 15 single clones selected for sequencing from each replicate sample.

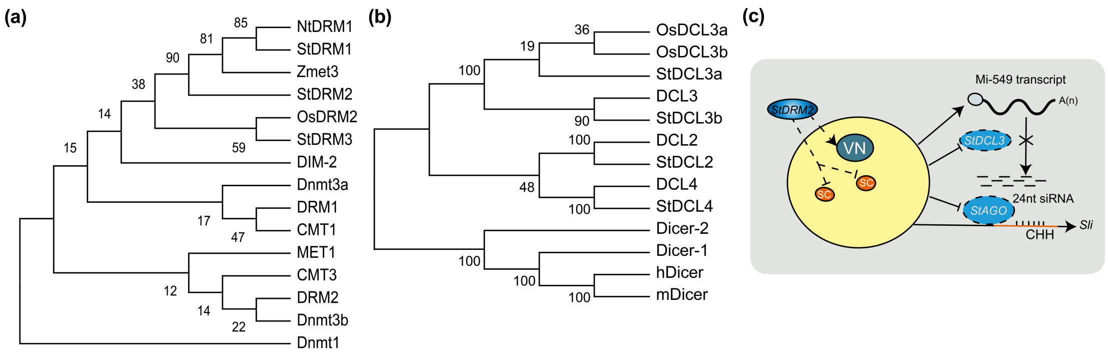

Fig. S5 RdDM related genes in potato was obtained by phylogenetic tree analysis and CHH methylation pattern in pollen. (a) phylogenetic relationships among DNA methyltransferases. Sequence data were obtained from the data base. (b) phylogenetic relationships among Dicer-like proteins (DCL). (e) CHH methylation pattern in mature potato pollen.

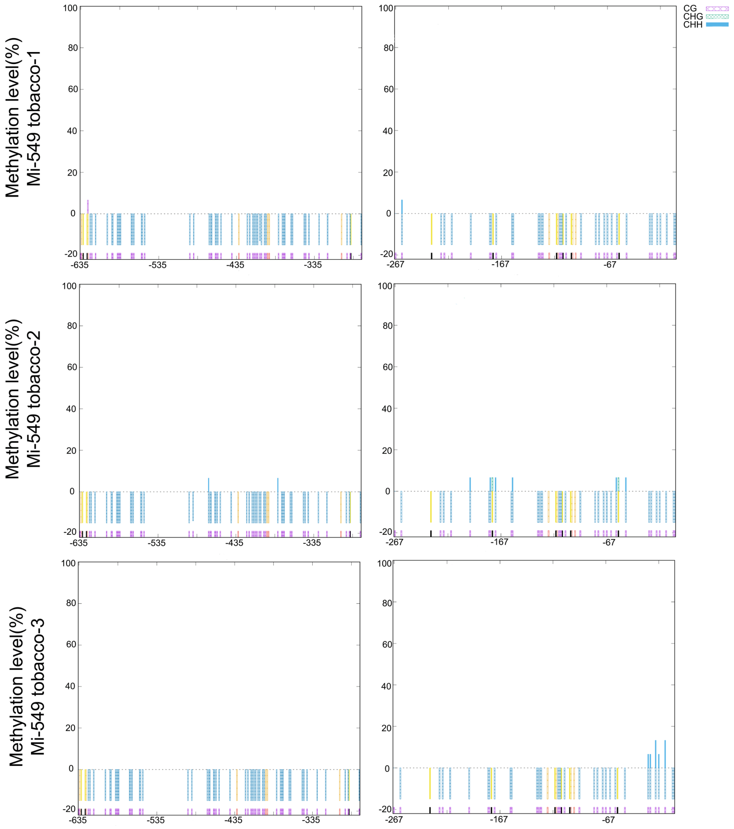

Fig. S6 Methylation level of Mi-549 in tobacco leaves. Cytosine methylation levels of Mi-549 and -86 bp regions and core promoter in M+ of tobacco LUC. The results of bisulfite analysis were analyzed using the program provided by Ravi LAB (https://katahdin.girihlet.com/kismeth/revpage.pl). The experimental procedure involved three independent biological replicates, and 15 single clones were selected from each replicate sample for sequencing.

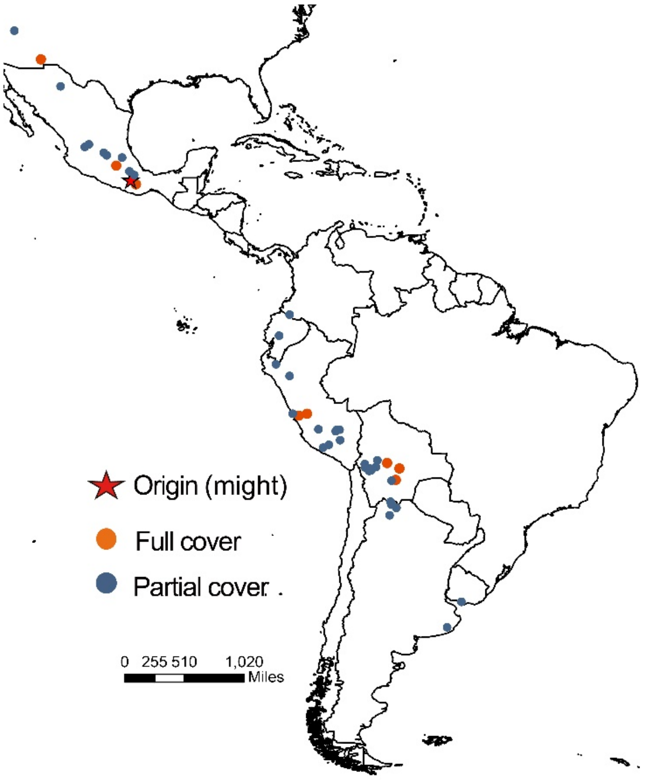

Fig. S7 Geographic distribution of germplasm with partial and complete Mi-549 insertions in the Americas. The distribution of germplasm with partial and complete insertions was mapped using a map of the Americas, incorporating the longitude and latitude coordinates of the collection sites provided on the potato germplasm website. The red five-pointed star denotes the germplasm *S. lesteri* (PG1010), where the Mi-549 insertion was first identified; orange dots indicate the locations with full coverage, while dark blue marks the locations of germplasm exhibiting partial coverage.

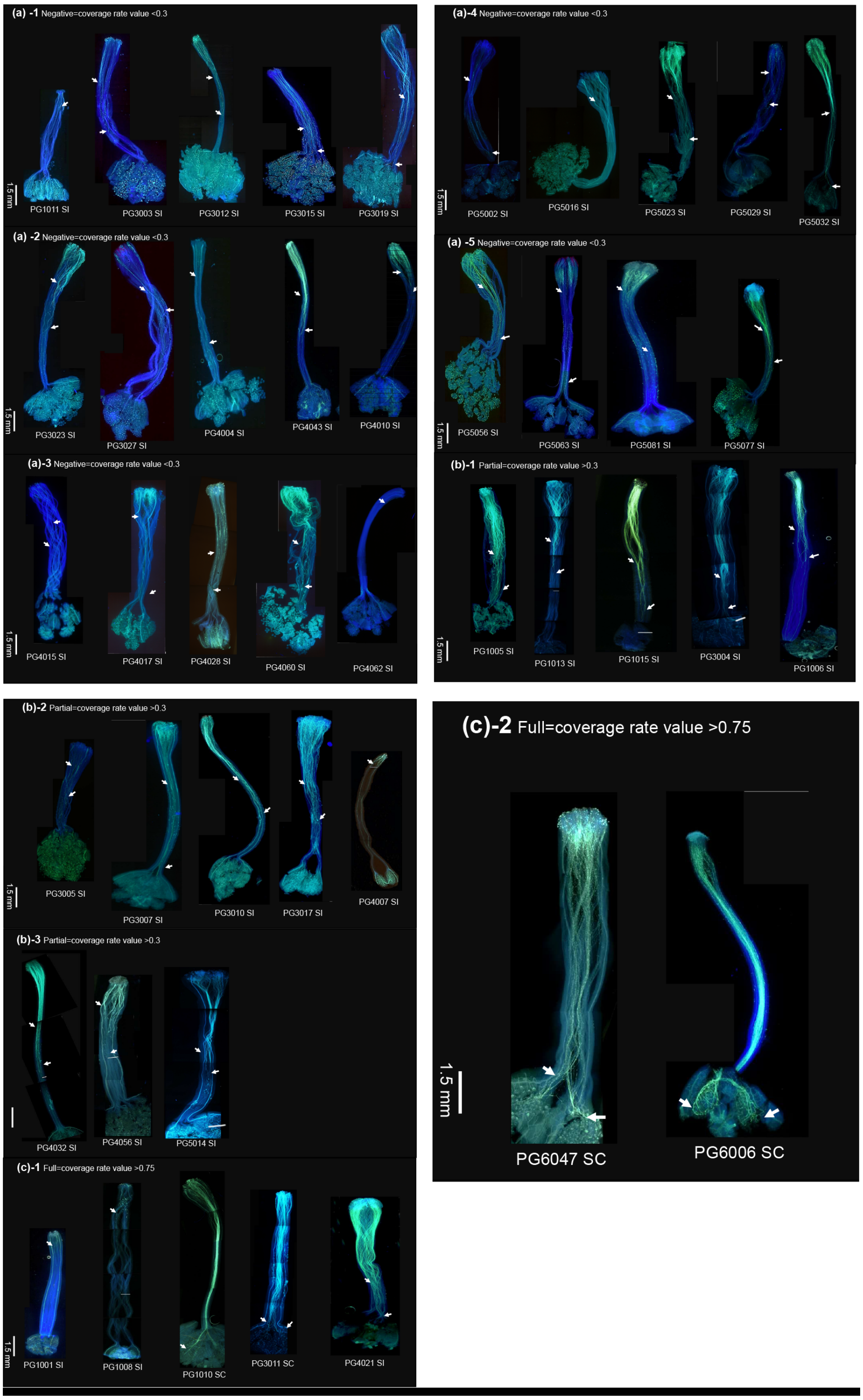

Fig. S8 Pollen tube elongation in 44 diploid potato accessions following self-pollination. Accessions from different clades were selected for self-pollination experiments, categorized by the Mi-549 insertion type in the *Sli* gene promoter region (a, negative = 24; b, partial = 13; c, full cover = 7). The arrow at the upper part of the style indicates the region where the majority of pollen tubes develop, while the arrow at the lower part of the style points to the location of the longest pollen tube growth.

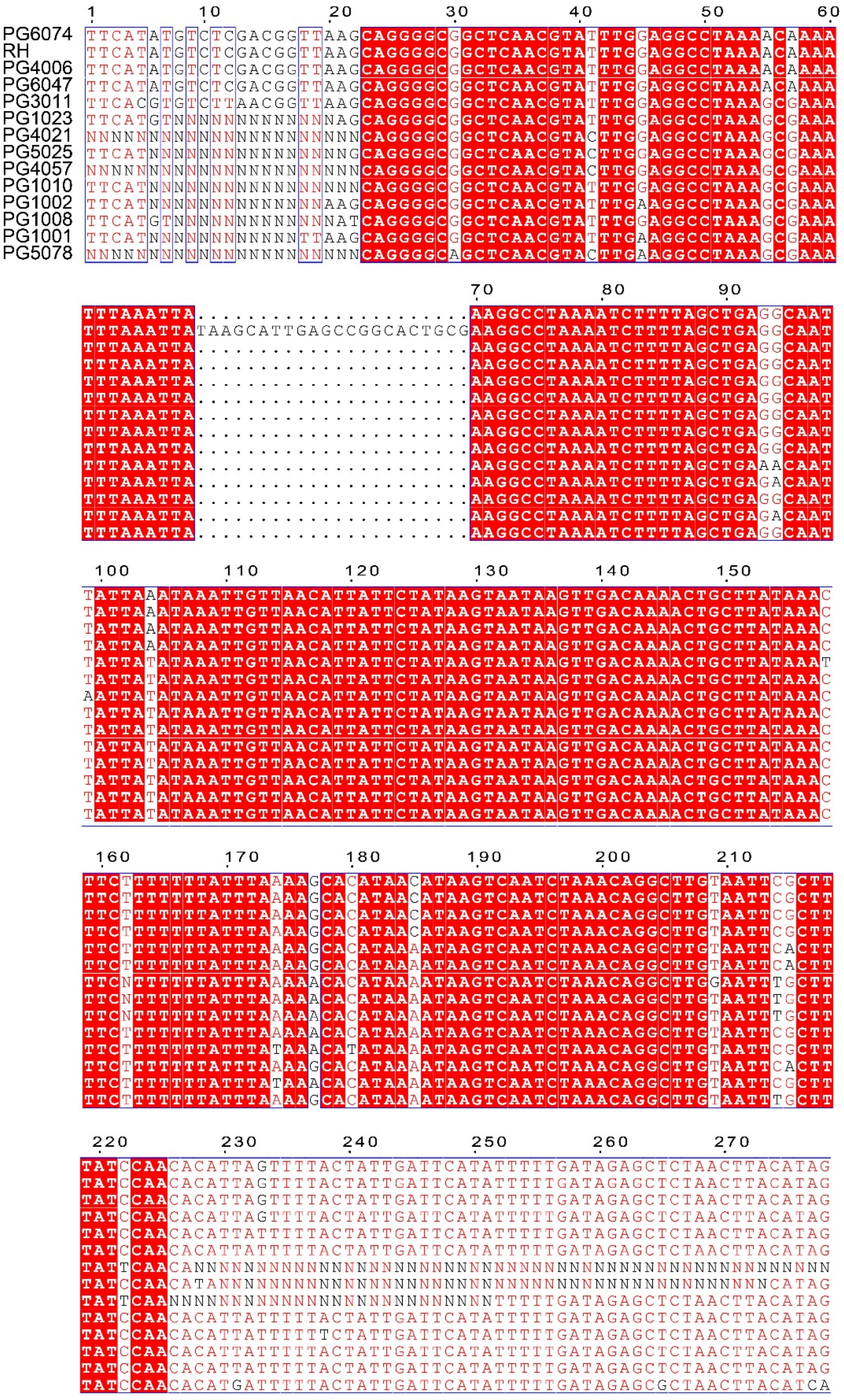

1 10 20 30 40 50 60
PG6074
RH
PG4006
PG6047
PG3011
PG1023
PG4021
PG5025
PG4057
PG1010
PG1002
PG1008
PG1001
PG5078
70 80 90
100 110 120 130 140 150
160 170 180 190 200 210
220 230 240 250 260 270

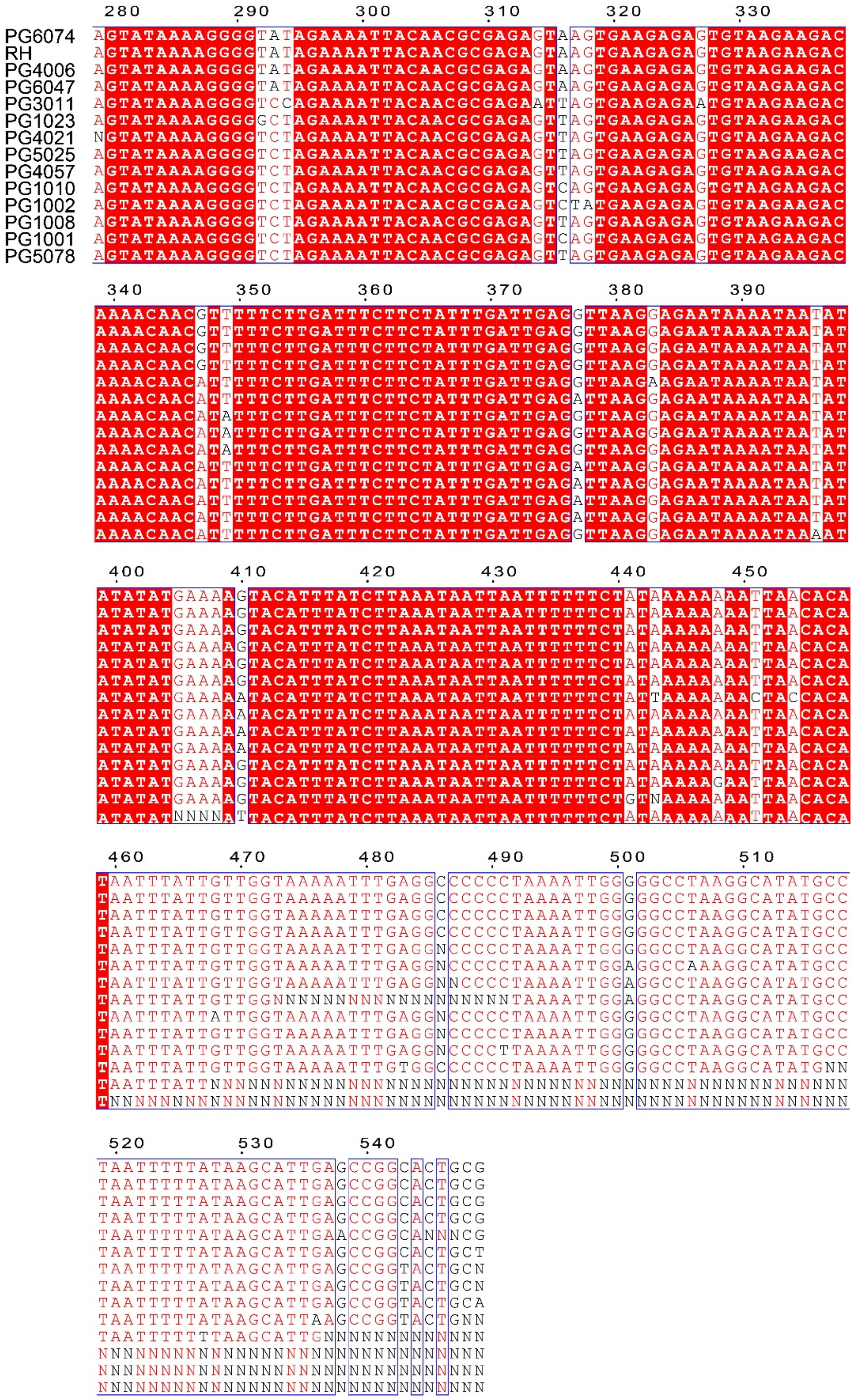

Fig. S9 Alignment of *Sli* promoter DNA sequence between the 13 accessions with full Mi-549 based on WGS data. The sequences extracted from WGS data, which full cover the *Sli* promoter region (-665 to +34 bp) of the germplasm.

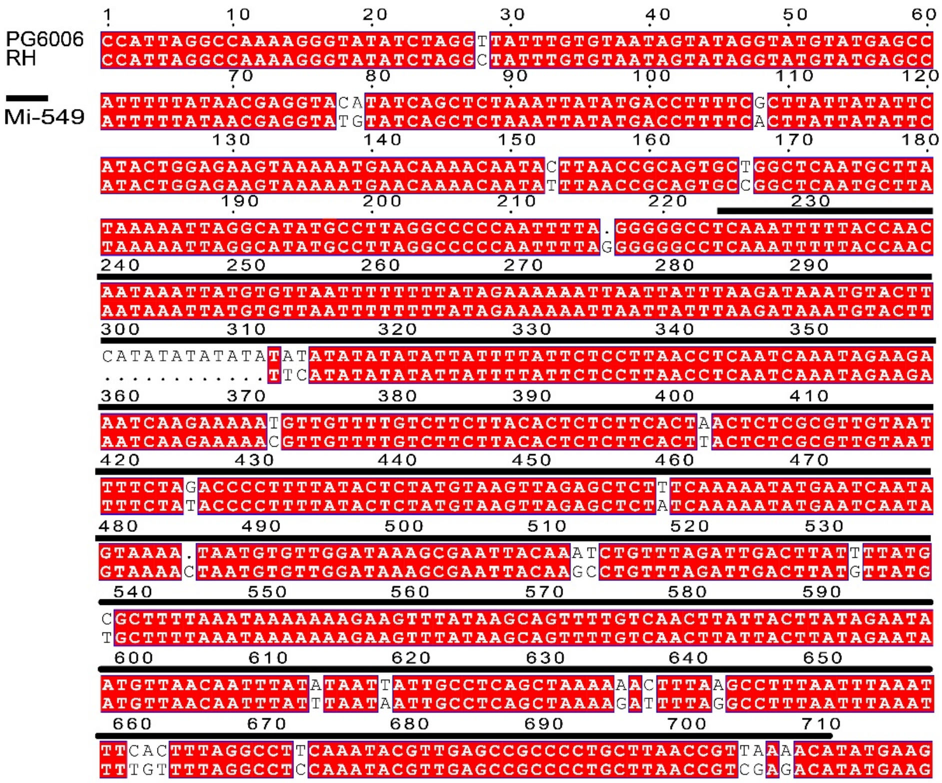

Fig. S10 Comparison of *Sli* promoter sequences between accessions PG6006 and RH. Amplification primers were designed based on the *Sli* promoter sequence of the RH germplasm, and the DNA sequence of PG6006 was utilized as a template for amplification using high-fidelity Taq enzyme. The amplified sequences were subsequently ligated into a TA cloning vector and sequenced for comparative analysis. The black line marks the Mi-549 insertion sequence.

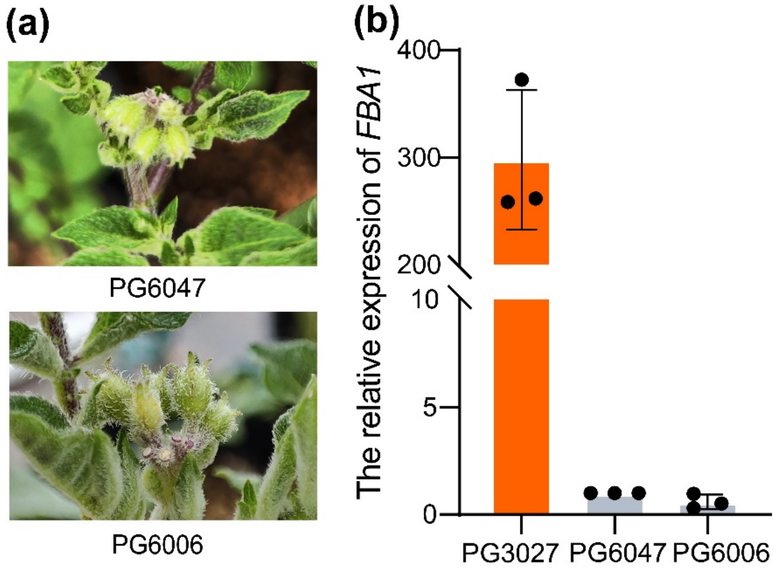

Fig. S11 The inbreeding depression gene *FBA1* affects abnormal flower development in PG6047 and PG6006. (a) Flowers with developmental failure in PG6006 and PG6047. (b) No

expression of the *FAB1* gene was detected in the non-developing flower buds compared to those that developed normally.

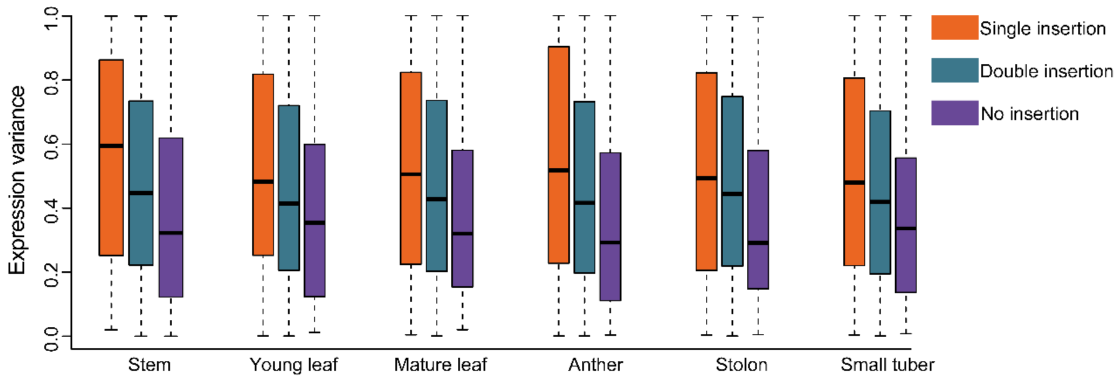

Fig. S12 Effect of MITE insertion in the promoter region on the expression variation of homologous genes in RH. The effect of MITE insertions on gene expression variation across different tissues (stem, young leaf, mature leaf, anther, stolon, and small tuber) was analyzed using RNA-seq data. Three insertion categories were examined: Single insertion (MITE inserted in the promoter region of one homeolog genes), Double insertion (MITE insertions present in both homologous genes promoter regions), and No insertion (no MITE insertions in either homologous genes promoter region).

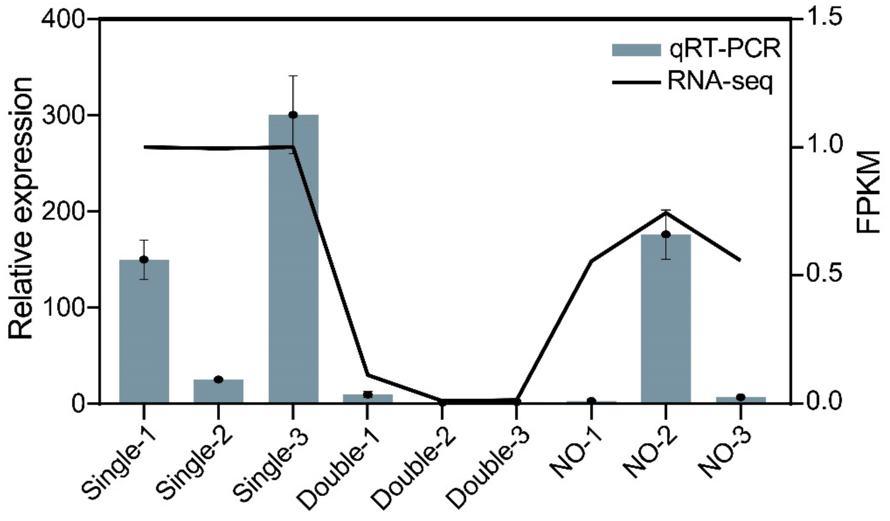

Fig. S13 Validation of the MITE insertion induced homologous gens expression difference using RNA-seq and qRT-PCR. Data are presented as means ± SD (n = 3 biologically independent replicates).

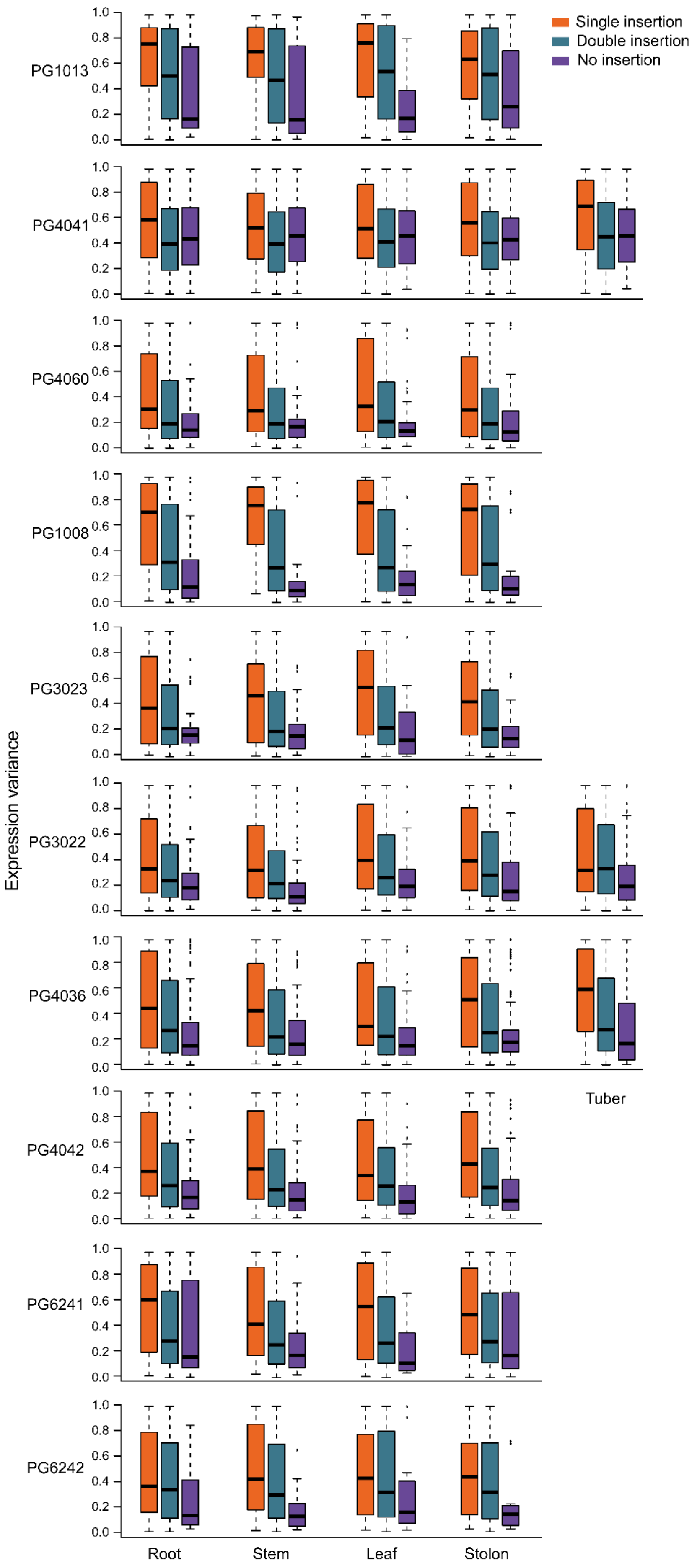

Single insertion
Double insertion
No insertion
PG1013
PG4041
PG4060
PG1008
PG3023
PG3022
PG4036
PG4042
PG6241
PG6242
Expression variance
1.0
0.8
0.6
0.4
0.2
0.0
Root
Stem
Leaf
Stolon
Tuber

Fig. S14 Effect of MITE insertion in the promoter region on homologous genes expression variation within the diploid potato genome. Three insertion categories were examined: Single insertion (MITE inserted in the promoter region of one homeolog genes), Double insertion (MITE insertions present in both homologous genes promoter regions), and No insertion (no MITE insertions in either homologous genes promoter region).
